# Identification of charged amino acids required for nuclear localization of human L1 ORF1 protein

**DOI:** 10.1186/s13100-019-0159-2

**Published:** 2019-05-06

**Authors:** B. T. Freeman, M. Sokolowski, A. M. Roy-Engel, M. E. Smither, V. P. Belancio

**Affiliations:** 1 Department of Structural and Cellular Biology, Tulane University School of Medicine, Tulane Cancer Center, Tulane Center for Aging, New Orleans, LA 70112 USA; 2 Department of Epidemiology, School of Public Health and Tropical Medicine, Tulane Cancer Center, Tulane University, New Orleans, Louisiana 70112 USA

**Keywords:** Retrotransposon, LINE-1, L1, ORF1p

## Abstract

**Background:**

Long Interspersed Element 1 (LINE-1) is a retrotransposon that is present in 500,000 copies in the human genome. Along with Alu and SVA elements, these three retrotransposons account for more than a third of the human genome sequence. These mobile elements are able to copy themselves within the genome via an RNA intermediate, a process that can promote genome instability. LINE-1 encodes two proteins, ORF1p and ORF2p. Association of ORF1p, ORF2p and a full-length L1 mRNA in a ribonucleoprotein (RNP) particle, L1 RNP, is required for L1 retrotransposition. Previous studies have suggested that fusion of a tag to L1 proteins can interfere with L1 retrotransposition.

**Results:**

Using antibodies detecting untagged human ORF1p, western blot analysis and manipulation of ORF1 sequence and length, we have identified a set of charged amino acids in the C-terminal region of ORF1p that are important in determining its subcellular localization. Mutation of 7 non-identical lysine residues is sufficient to make the resulting ORF1p to be predominantly cytoplasmic, demonstrating intrinsic redundancy of this requirement. These residues are also necessary for ORF1p to retain its association with KPNA2 nuclear pore protein. We demonstrate that this interaction is significantly reduced by RNase treatment. Using co-IP, we have also determined that human ORF1p associates with all members of the KPNA subfamily.

**Conclusions:**

The prediction of NLS sequences suggested that specific sequences within ORF1p could be responsible for its subcellular localization by interacting with nuclear binding proteins. We have found that multiple charged amino acids in the C-terminus of ORF1p are involved in ORF1 subcellular localization and interaction with KPNA2 nuclear pore protein. Our data demonstrate that different amino acids can be mutated to have the same phenotypic effect on ORF1p subcellular localization, demonstrating that the net number of charged residues or protein structure, rather than their specific location, is important for the ORF1p nuclear localization. We also identified that human ORF1p interacts with all members of the KPNA family of proteins and that multiple KPNA family genes are expressed in human cell lines.

**Electronic supplementary material:**

The online version of this article (10.1186/s13100-019-0159-2) contains supplementary material, which is available to authorized users.

## Background

Long interspersed element 1 (L1) is a non-long terminal repeat retrotransposon that accounts for approximately 21% of the human genome [1]. L1 has colonized the genome for millions of years via an autonomous copy-and-paste mechanism [2]. L1 is also responsible for the mobilization of non-autonomous retrotransposons such as Alu and SVA elements [3]. Although L1 has been found in an evolutionarily diverse range of species, its proteins show conservation of specific functional domains and residues [4]. While the majority of L1 insertions are severely 5′ truncated, an estimated 80–100 L1 elements per genome are full-length, thus still capable of independent retrotransposition [5].

The full length L1 is a 6 kb retrotransposon that consists of a 5’UTR, ORF1, ORF2, and a 3′ UTR [6]. The 5′ UTR includes an internal polII promoter and the 3′ UTR contains a terminal poly-a sequence [7, 8]. ORF1 encodes ORF1 protein (ORF1p), a homotrimeric protein with nucleic acid chaperone activities [9, 10]. ORF2 encodes ORF2 protein (ORF2p), which contains reverse transcriptase and endonuclease activities necessary for insertion of the element [11, 12].

ORF1p and ORF2p interact with their own mRNA in cis to form a ribonucleoprotein (RNP) particle in the cytoplasm [6, 13–15]. This acts as a likely intermediate for retrotransposition. How the L1 RNP accesses genomic DNA is poorly understood. Published work supports the possibility of both passive and active nuclear transport mechanisms [16–22]. Once access to the genomic DNA is gained, ORF2p functions to copy the L1 mRNA for integration into a new location in the host genome, a process termed target-primed reverse transcription (TPRT) [23, 24].

Recent research has indicated that retrotransposition events can promote genomic instability relevant to cancer origin and/or progression [25–27]. Global L1 DNA hypomethylation, along with a corresponding increase in L1 expression, has been reported in many cancer types as compared to benign tissue [28–32]. An increase in overall ORF1p expression has been detected in a wide variety of patient tumor types, including bladder urothelial carcinoma [33], Barrett’s esophagus [34], and others [35–37]. Nuclear ORF1p, specifically, is seen to correlate with poor prognostic outcomes in breast cancer patients [38, 39]. Consistent with in vivo findings, in vitro studies have shown that overexpressed untagged ORF1p localizes to both the nucleus and cytoplasm in human cell lines [40–44].

**Fig. 1:**
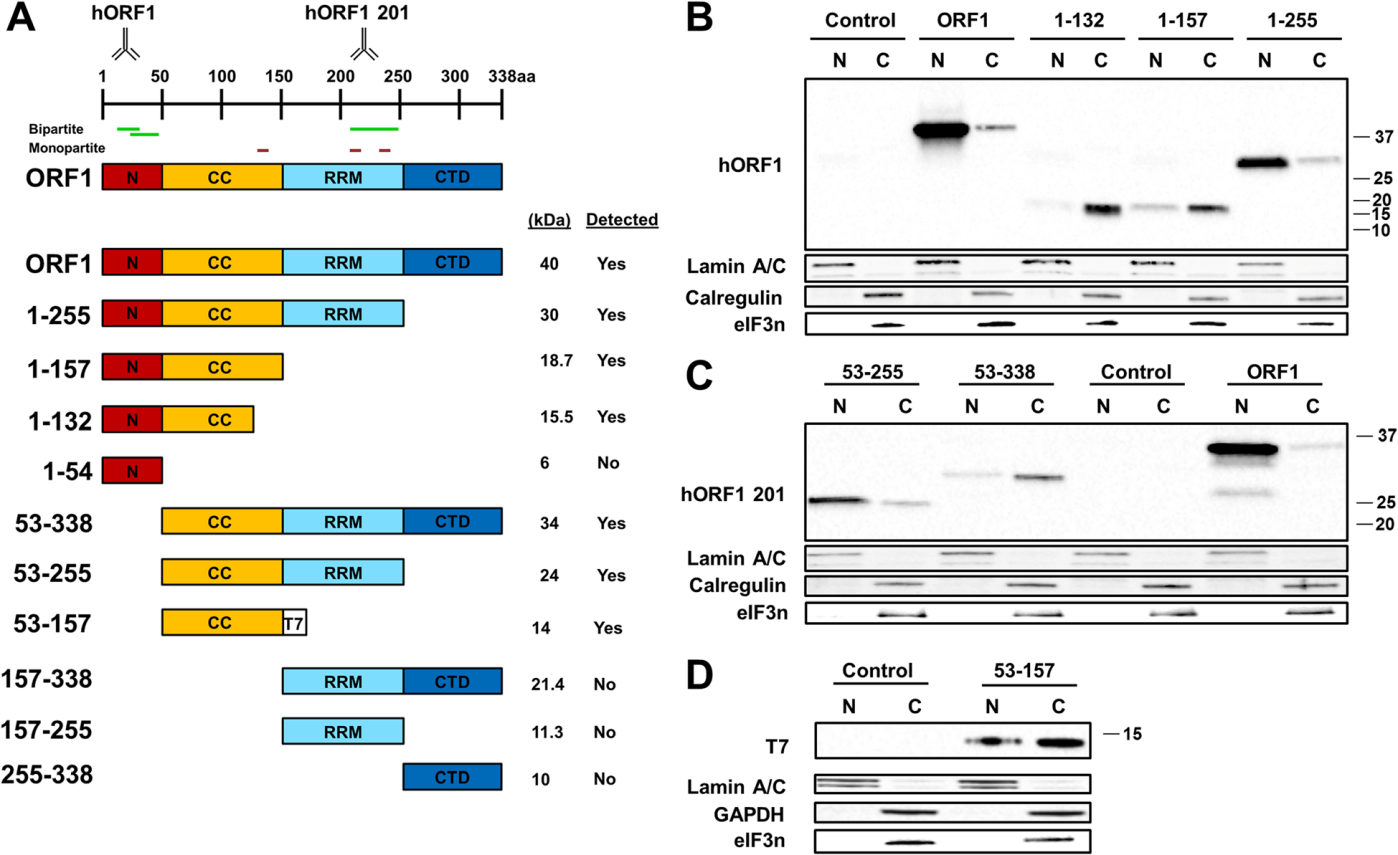
Truncated ORF1 proteins require the RRM domain for their nuclear localization. **a** (top) ORF1 domains are indicated as an N-terminal domain (N), a coiled-coil domain (CCD), and RNA recognition motif (RRM) and a C-terminal domain (CTD). Positions of domains shown are approximate. The antibody symbols (name above) denote approximate location of antibodies on the ORF1p. Putative bipartite nuclear localization signal (Bipartite) is shown in green and putative monopartite nuclear localization signal (monopartite) is shown in brown. (bottom) Schematic of the ORF1 truncation approach. The expected molecular weight of each construct is listed to the right as well as detection of the protein generated from the construct (Detected column). The numbers listed on the left represent the position of the site of truncation based on a full-length ORF1p. The 53–157 construct has a T7 tag. **b** Western blot analysis of truncated human ORF1 transiently transfected in HeLa cells. Proteins are separated into nuclear (N) and cytoplasmic (C) fractions. Human ORF1 protein was detected with human-specific ORF1 polyclonal antibodies (hORF1). Calregulin (cytoplasmic marker) and Lamin A/C (nuclear marker) are used as loading and cell fractionation controls and eIF3 is used to control for cytoplasmic stress granules. Positions of molecular markers are indicated on the right in kDa. Control indicates cells transfected with empty plasmid. **b** Western blot analysis of truncated human ORF1 transiently transfected in HeLa cells. Proteins are separated into nuclear (N) and cytoplasmic (C) fractions. Human ORF1 protein was detected with human-specific ORF1 polyclonal antibodies (hORF1 201). Calregulin (cytoplasmic marker) and Lamin A/C (nuclear marker) are used as loading and cell fractionation controls. Positions of molecular markers are indicated on the right in kDa. Control indicates cells transfected with empty plasmid. **d** Western blot analysis of truncated human ORF1 transiently transfected in HeLa cells. Proteins are separated into nuclear (N) and cytoplasmic (C) fractions. Human ORF1 protein was detected with T7 tag polyclonal antibodies (T7). GAPDH (cytoplasmic marker) and Lamin A/C (nuclear marker) are used as loading and cell fractionation controls. Positions of molecular markers are indicated on the right in kDa. Control indicates cells transfected with empty plasmid

ORF1p is a 338 amino acid protein that is necessary for L1 retrotransposition [13, 45]. It contains four domains (Fig. 1a). The N-terminal domain (NTD, AAs 1–54) is intrinsically disordered and has a putative role in RNA binding [9]. It also contains two conserved residues, phosphorylation of which is necessary for retrotransposition in cultured cells [46]. The coiled coil domain (CCD, AAs 55–157) contains 14 heptad repeats that facilitate ORF1p trimerization [9, 47]. The RNA recognition motif (RRM, AAs 157–255) and the C-terminal Domain (CTD, AAs 256–338) bind directly to RNA [9]. ORF1p binds both DNA and RNA in a nonsequence-specific manner, showing a preference for single stranded over double stranded nucleic acids [48, 49]. ORF1p also has chaperone activities involving melting, annealing, and strand exchange of nucleic acids [50, 51]. After translation, ORF1p trimers bind to their parental mRNA [6], potentially providing stability to the RNP as it continues through the retrotransposition process. Each ORF1p trimer occupies approximately 50 nucleotides during RNP formation [52], indicating that ORF1p could be abundant in RNP complexes. Purification of L1 RNPs confirmed higher content of ORF1p compared to the amount of ORF2p [22]. Although there is a strong cis preference for L1 proteins to associate with their nascent mRNA to form RNPs [15], ORF1p is also used in trans during SVA mobilization [53] and has been shown to improve Alu retrotransposition [54]. In vitro and cell culture-based analyses have shown that ORF1p molecules made from different mRNAs can associate in trans and that certain C-terminally truncated ORF1p constructs can suppress L1 retrotransposition in vitro [41]. Conserved groups of amino acids in the CTD have been previously shown to be necessary for L1 retrotransposition, as mutation of these amino acids into alanine reduces L1 retrotransposition to < 1% of wild type [9, 11, 55].

Subcellular localization of L1 proteins during the L1 replication cycle or non-specific ORF1p complexes present during ectopic expression of ORF1p is poorly understood as conflicting results regarding the requirement of cellular division for L1 retrotransposition have been reported [16–22]. Recent studies indicate that L1 may access the nucleus passively during breakdown of the nuclear membrane [16]. However, reports that L1 retrotransposition occurs in nondividing cells support the potential role of active transport for L1 and ORF1p, which is often used as a proxy for L1 RNPs [17, 18]. This is further supported by the findings that L1 ORF1p associates with nuclear pore proteins [19, 22, 56] and by the recent finding that ORF1p nuclear localization is not affected by cell cycle arrest [44]. Recent findings show the existence of distinct nuclear and cytoplasmic RNP complexes with ORF1p-rich RNPs being found predominantly in the cytoplasmic fraction. However, previous studies in HeLa cells show that overexpression of flag-tagged ORF2p is only detected in the nucleus when co-expressed with ORF1p, indicating a role for ORF1p on L1 RNP subcellular localization [57]. Studies done in 143B TK-cells, however, only show a slight increase in nuclear flag-tagged ORF2p when co-expressed with ORF1p [58]. Thus, it remains unclear whether active nuclear import of ORF1p (and possibly, by extension, the L1 RNP) occurs during retrotransposition and whether cell-type specific differences in regards to ORF1p localization exist. Therefore, determining requirements for subcellular localization of L1 proteins remains an unresolved, but important, problem because of its clinical significance and relevance to basic L1 (and its parasites - SINEs and SVA elements) biology.

Although the presence of a functional nuclear localization signal (NLS) in the human ORF1p has not been documented, ORF1p in a non-LTR retrotransposon, SART1 from *Bombyx mori*, contains a NLS required for the nuclear import of its RNP [59]. A putative NLS was predicted in the human L1 ORF2p, but mutagenesis studies of this motif have shown no significant effect on localization of the ORF2p fragments containing this sequence [60] A nucleolar localization signal (NoLS), however, has been identified in ORF2p [58]. Mass spectrometry analysis of flag-tagged human ORF1p identified association with nuclear pore proteins KPNA2 and KPNB1 in HEK293T cells [56], suggesting that this protein contains a functional NLS. KPNA2 typically binds to a protein’s NLS and serves as an adaptor for KPNB1 association, which provides the import activity of the complex [61, 62]. Multiple classes of NLSs have been identified. Canonical (or classical) NLSs are specific sequences that bind to KPNA family of proteins. These NLSs can be mono- or bipartite [63]. Bipartite NLSs are typically comprised of two imperfect monopartite NLSs separated by 10–12 amino acids [61]. KPNA family proteins have both major and minor binding grooves. Monopartite NLSs bind to just one of the two grooves. Bipartite NLSs bind to both grooves [63]. Another class are noncanonical NLSs that, instead of being specific sequences, are composed of charged, basic amino acids (typically arginines or lysines) that bind to the nuclear pore proteins. All noncanonical NLSs bind to only the minor binding groove of KPNA [64, 65]. Several NLS prediction programs have been developed using yeast and/or human proteins and various algorithms to identify putative NLSs [66–68]. While the previously published mass spectrometry analysis strongly supports the association of ORF1p with the nuclear import complex [56], the sequence requirements within ORF1p that facilitate this association remain unknown.

Many experimental studies of ORF1p localization have previously relied on the use of tags for detection [40, 41, 56, 57, 69, 70] because, in the past, it was the only approach available at the time. GFP-tagged ORF1p is detected primarily in the cytoplasm by IHC, and subcellular localization of this tagged protein has been shown to be affected by truncations of ORF1p [69]. However, the GFP-tag has since been shown in some instances to interfere with authentic subcellular localization of ORF1p and other proteins (Author’s response of [16]) [71]. ,Using ORF1p-specific antibodies, more recent publications reported direct detection of untagged human (transiently expressed or endogenous) ORF1p providing more biologically relevant findings for the purposes of localization studies [39–41, 44, 70, 72].

Here, using untagged human ORF1p, western blot approach, and cultured human cells, we demonstrate that the RRM domain of ORF1p is a crucial determinant for the nuclear localization of the full-length and truncated human ORF1 proteins. We utilized mutation and deletion analyses to identify specific amino acids that are necessary for ORF1p nuclear localization and association with the KPNA family of nuclear pore proteins. Using co-IP approach, we demonstrate that human ORF1p associates with all members of the KPNA family tested in this study. These findings support the presence of a noncanonical NLS and redundancy in the ORF1p interaction with the nuclear pore machinery.

## Results

### N-terminal and C-terminal truncations of ORF1p require RRM for nuclear localization in transiently transfected cells

We used human ORF1p sequence and the following NLS prediction programs: Machine Learning and Evolution Laboratory (MLEG), cNLS Mapper, or NLStradamus [66–68] to identify putative nuclear localization signals (NLSs). Approximate locations of putative Bipartite (green) and Monopartite (brown) nuclear localization signals are illustrated in Fig. 1a. The bipartite NLSs are located from amino acids 6 to 34, 19 to 48, and 202 to 237 and the monopartite NLSs are located from amino acids 135 to 138, 210 to 215, and 235–238 (Fig. 1a).

We used deletion analysis to determine which NLS-containing domains are necessary for nuclear localization of untagged human ORF1p. The N-terminal and C-terminal truncated ORF1 proteins were generated based on the breakpoints of previously identified functional domains, as well as position of putative NLSs (Fig. 1a). Most of the resulting truncated ORF1 proteins were stable as they were detectable by either custom ORF1p specific antibodies labeled hORF1 or hORF1 201 (Fig. 1a-c) [40, 41, 72] or T7-specific antibodies (Fig. 1a and d). However, many of the smaller ORF1p fragments, such as ORF1 1–54 that contains only the NTD, and all N-terminally truncated proteins lacking the CCD of ORF1p were undetectable under used experimental conditions.

**Fig. 2:**
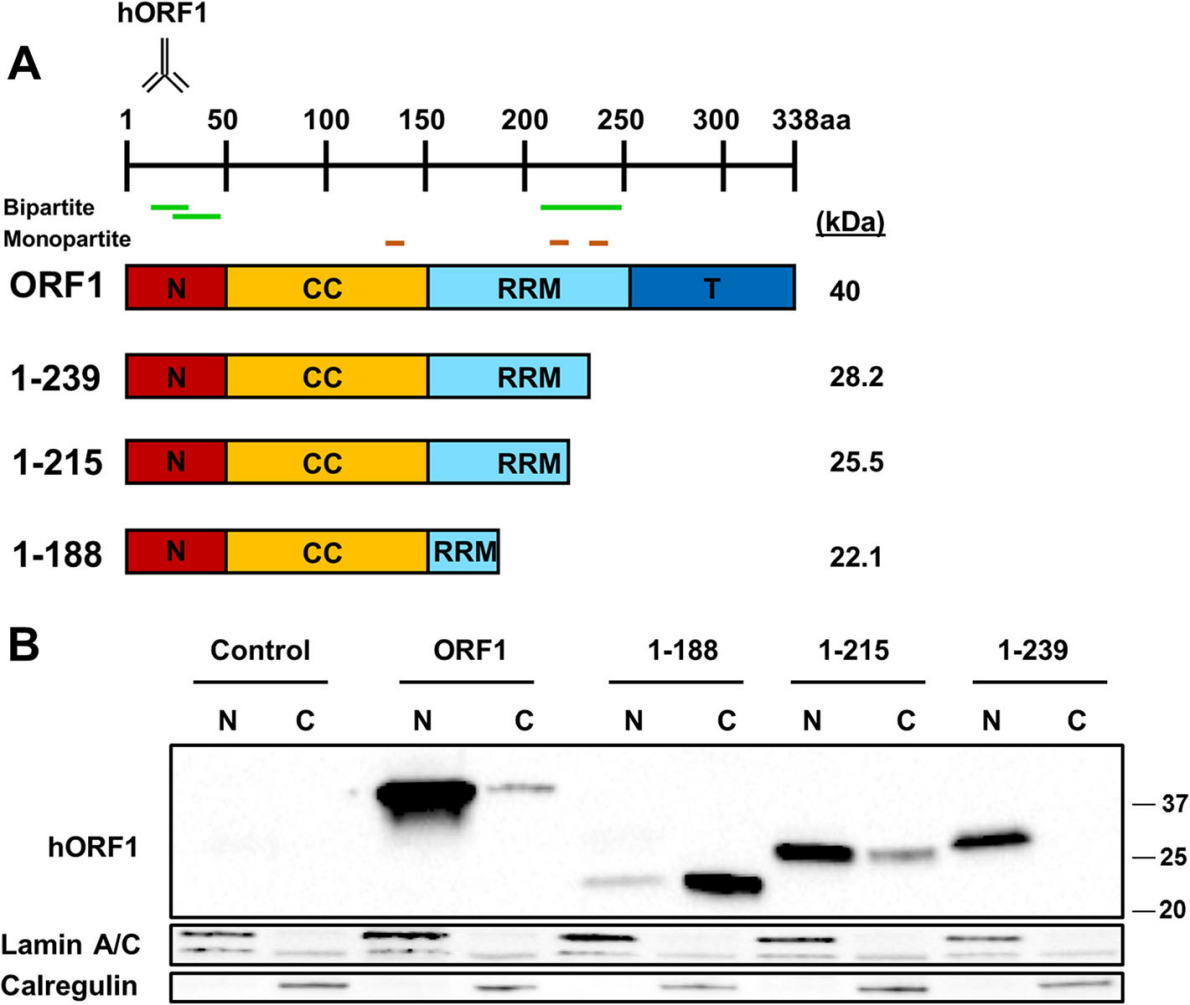
The nuclear localization of the truncated ORF1 proteins is narrowed down to a 29 amino acid region within the RRM domain. **a** (top) ORF1 domains are indicated as an N-terminal domain (N), a coiled-coil domain (CCD), and RNA recognition motif (RRM) and a C-terminal domain (CTD). Positions of domains shown are approximate. The antibody symbol (name above) denotes approximate location of the antibody on the ORF1p. Putative bipartite nuclear localization signal (Bipartite) is shown in green and putative monopartite nuclear localization signal (monopartite) is shown in brown. **(bottom)** Schematic of the truncated and full-length ORF1 constructs. The expected molecular weight of each construct is listed to the right. The numbers listed on the left represent the position of the site of truncation based on a full-length ORF1p. **b** Western blot analysis of truncated human ORF1 transiently transfected in HeLa cells. Proteins are separated into nuclear (N) and cytoplasmic (C) fractions. Human ORF1 protein was detected with human-specific ORF1 polyclonal antibodies (hORF1). Calregulin (cytoplasmic marker) and Lamin A/C (nuclear marker) are used as loading and cell fractionation controls. Positions of molecular markers are indicated on the right in kDa. Control indicates cells transfected with empty plasmid

**Fig. 3:**
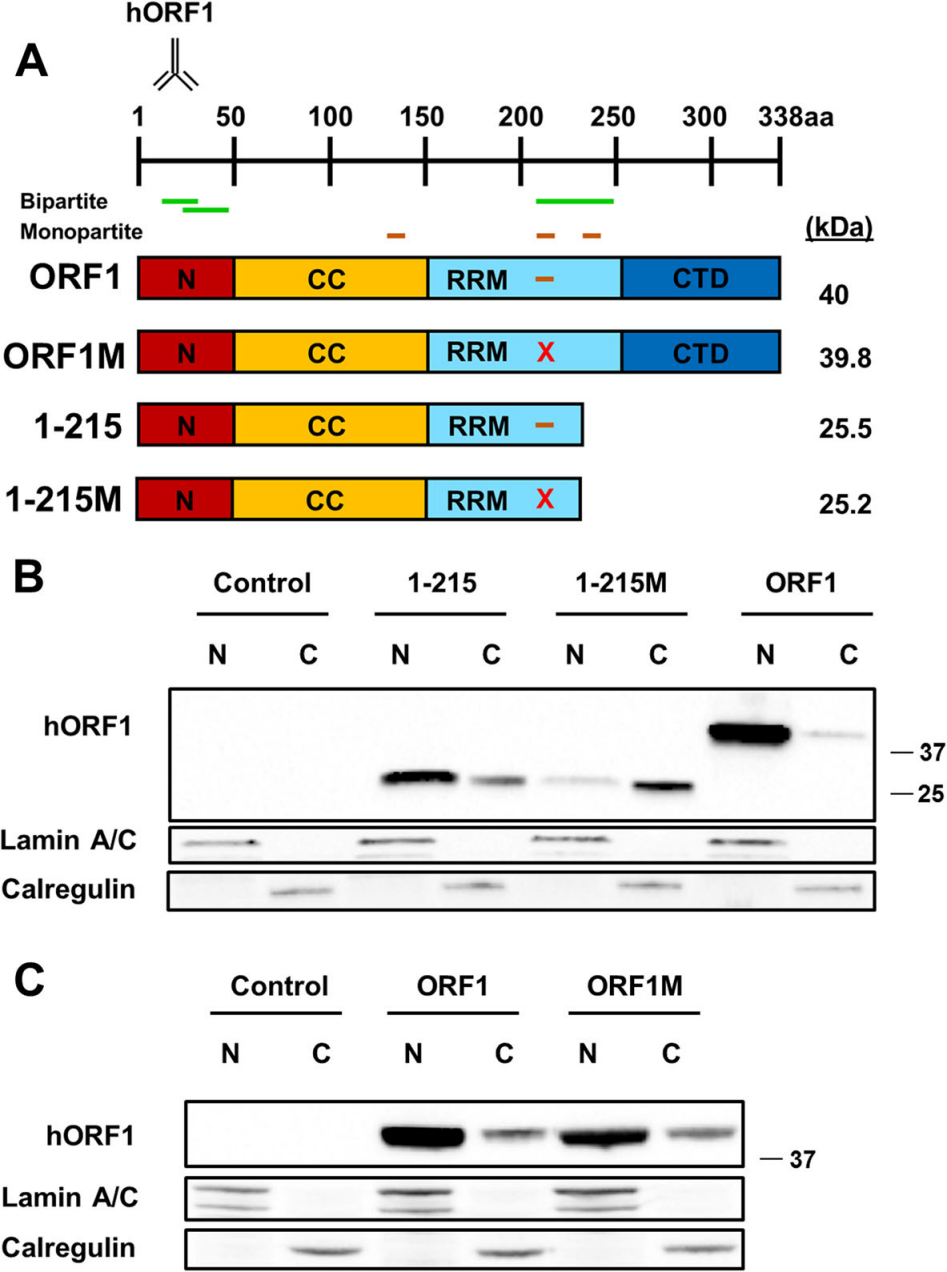
Mutation of the putative nuclear localization signal results in a shift of the truncated ORF1p into the cytoplasmic fraction. **a** (top) ORF1 domains are indicated as an N-terminal domain (N), a coiled-coil domain (CCD), and RNA recognition motif (RRM) and a C-terminal domain (CTD). Positions of domains shown are approximate. The antibody symbol (name above) denotes approximate location of the antibody on the ORF1p. Putative bipartite nuclear localization signal (Bipartite) is shown in green and putative monopartite nuclear localization signal (monopartite) is shown in brown. The red “X” (X) denotes the mutation of the ORF1 R206/R210/R211 amino acid positions into alanine residues. **(bottom)** Schematic of the truncated and full-length ORF1 constructs. The expected molecular weight of each construct is listed to the right. The numbers listed on the left represent the position of the site of truncation based on a full-length ORF1p. **b** Western blot analysis of truncated and full-length human ORF1 transiently transfected in HeLa cells. Proteins are separated into nuclear (N) and cytoplasmic (C) fractions. Human ORF1 protein was detected with human-specific ORF1 polyclonal antibodies (hORF1). Calregulin (cytoplasmic marker) and Lamin A/C (nuclear marker) are used as loading and cell fractionation controls. Positions of molecular markers are indicated on the right in kDa. Control indicates cells transfected with empty plasmid. **c** Western blot analysis of full-length human ORF1 transiently transfected in HeLa cells. Proteins are separated into nuclear (N) and cytoplasmic (C) fractions. Human ORF1 protein was detected with human-specific ORF1 polyclonal antibodies (hORF1). Calregulin (cytoplasmic marker) and Lamin A/C (nuclear marker) are used as loading and cell fractionation controls. Positions of molecular markers are indicated on the right in kDa. Control indicates cells transfected with empty plasmid

**Fig. 4:**
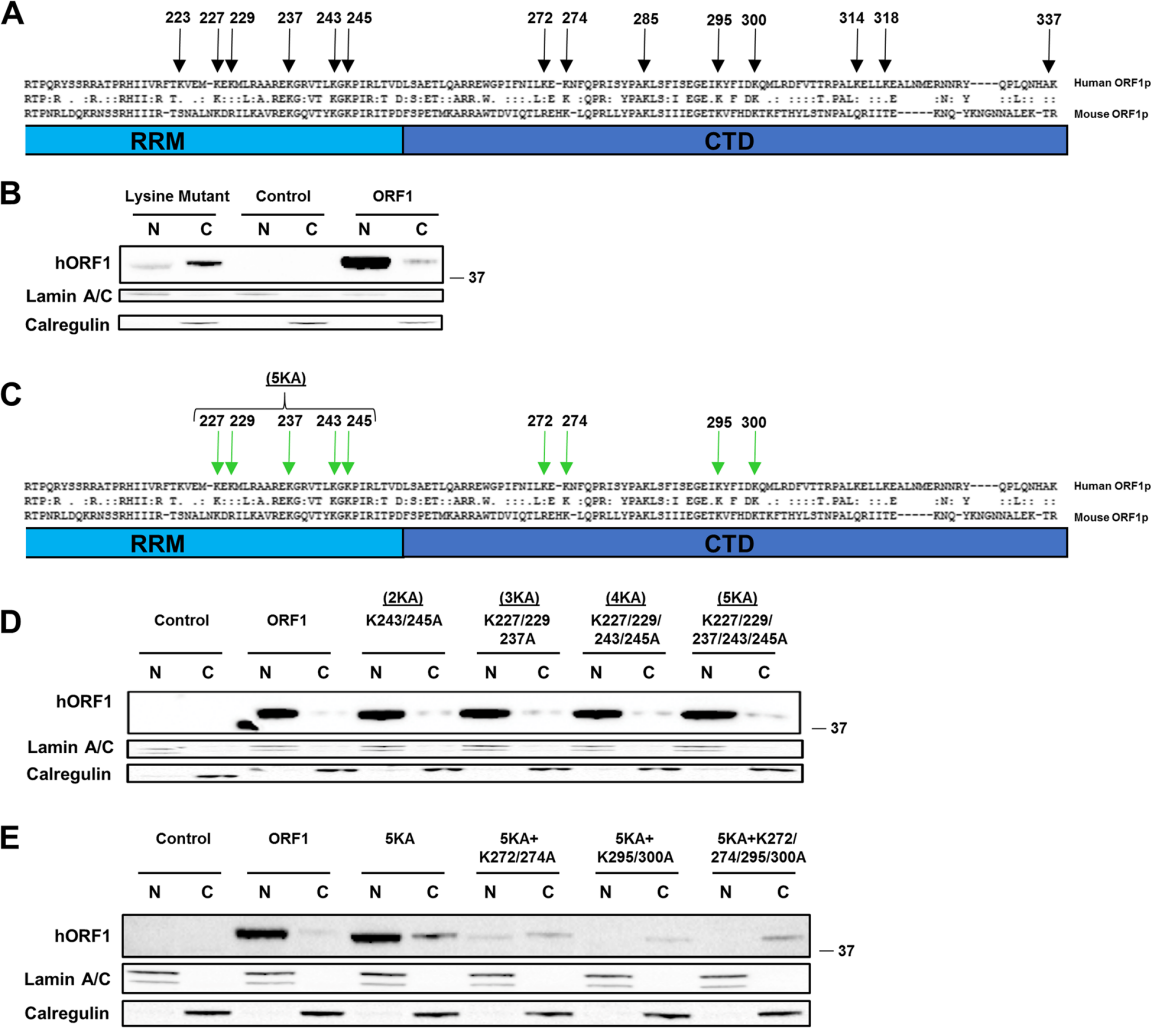
Mutation of charged residues in the RRM and CTD of the full-length ORF1 results in a shift of the truncated ORF1p into the cytoplasmic fraction. **a** Schematic of part of the RNA recognition motif (RRM) and C-terminal domain (CTD) of ORF1. Positions of domains shown are approximate. Human ORF1p (top) is aligned to mouse ORF1p (bottom) using Lipman-Pearson method. Black arrows denote the amino acid position of the lysine residues that were mutated to alanine residues in the human ORF1. The lysine mutant contains all 14 lysine residues (all black arrows) mutated to alanine residues. **b** Western blot analysis of full-length human ORF1 transiently transfected in HeLa cells. Proteins are separated into nuclear (N) and cytoplasmic (C) fractions. Human ORF1 protein was detected with human-specific ORF1 polyclonal antibodies (hORF). Calregulin (cytoplasmic marker) and Lamin A/C (nuclear marker) are used as loading and cell fractionation controls. Positions of molecular markers are indicated on the right in kDa. Control indicates cells transfected with empty plasmid. **c** Schematic for **(d)** and **(e)**. Green arrows denote the amino acid position of the lysine residues that were mutated to alanine residues in the human ORF1. Human ORF1p (top) is aligned to mouse ORF1p (bottom) using Lipman-Pearson method. Black arrows denote the amino acid position of the lysine residues that were mutated to alanine residues in the human ORF1. “(5KA)” denotes a construct in which the five residues listed (227,229, 237, 243 and 245) were mutated from lysine to alanine residues. **d** Western blot analysis of full-length human ORF1 transiently transfected in HeLa cells. Proteins are separated into nuclear (N) and cytoplasmic (C) fractions. Human ORF1 protein was detected with human-specific ORF1 polyclonal antibodies (hORF1). Calregulin (cytoplasmic marker) and Lamin A/C (nuclear marker) are used as loading and cell fractionation controls. Positions of molecular markers are indicated on the right in kDa. Control indicates cells transfected with empty plasmid. “(5KA)” denotes a construct in which the five residues (227,229, 237, 243 and 245) were mutated from lysine to alanine residues (227,229, 237, 243 and 245). **e** Western blot analysis of full-length human ORF1 transiently transfected in HeLa cells. Proteins are separated into nuclear (N) and cytoplasmic (C) fractions. Human ORF1 protein was detected with human-specific ORF1 polyclonal antibodies (hORF1). Calregulin (cytoplasmic marker) and Lamin A/C (nuclear marker) are used as loading and cell fractionation controls. Positions of molecular markers are indicated on the right in kDa. Control indicates cells transfected with empty plasmid. “(5KA)” denotes a construct in which the five residues (227,229, 237, 243 and 245) were mutated from lysine to alanine residues (227,229, 237, 243 and 245) in addition, other residues that were also mutated in the construct are listed above each lane

This deletion analysis revealed that the subcellular distribution of some of the truncated ORF1 proteins differed from that of the full-length ORF1p. Our method of separating the nuclear and cytoplasmic fractions has previously shown that calregulin (endoplasmic reticulum marker) affiliates with the cytoplasmic fraction [40], which can also be seen in this study (Figs. 1, 2, 3, 4). Additionally, eIF3, a component of cytoplasmic stress granules [73, 74], is detected in the cytoplasmic fraction. The 1–132 protein containing the NTD and part of the CCD was detected predominantly in the cytoplasmic fraction (Fig. 1a and b) [41]. A noticeable increase in their nuclear localization was observed for the 1–157 and 1–255 proteins, the latter of which was detected almost exclusively in the nuclear fraction similar to the full-length ORF1p (Fig. 1a and b). To test the contribution of the NTD to this pattern of subcellular localization, a 53–255 construct was generated (Fig. 1a). Its subcellular localization was identical to that of the 1–255 protein (Fig. 1b and c). However, an N-terminally truncated ORF1 protein lacking the NTD, 53–338, was detected more strongly in the cytoplasmic compared to the nuclear fraction (Fig. 1a and c, Additional file 1: Figure S1). Similarly, an ORF1 protein containing only the CCD was detected more efficiently in the cytoplasmic fraction (Fig. 1a and d, 53–157). In all cases, ORF1p fragments cannot efficiently localize to the nucleus without the RRM domain. These findings suggest that the RRM domain may contain sequences influencing subcellular distribution of human ORF1p (Fig. 1b).

### Truncated and full-length ORF1 proteins have different sequence requirements for their subcellular localization

To identify the sequences dictating the varying subcellular localization of the truncated ORF1p (Fig. 1a), we generated a subset of C-terminally truncated ORF1 proteins containing different length of the RRM domain in order to determine whether the putative NLSs predicted within the RRM are involved in subcellular localization of these proteins (Fig. 2a). The two putative monopartite NLSs are located between AAs 210–215 and 235–238 and overlap with the predicted bipartite NLS that is located between AAs 202–237. Western blot analysis of the truncated proteins shows that the removal of the monopartite pNLS present within aa 235–238 resulted in minimal change in subcellular localization of the 1–215 protein compared to the 1–239 protein (Fig. 2b, 1–215 versus 1–239). However, removal of the RRM sequences containing pNLSs resulted in predominantly cytosolic localization of the 1–188 protein (Fig. 2b). As partial removal of the putative bipartite and one of the predicted monopartite NLSs did not have any effect on subcellular localization of the 1–239 protein compared to the full-length ORF1p, we concluded that theses sequences are not functional NLSs.

To test the contribution of the pNLS at AAs 210–215 to the observed shift in localization, we mutated both arginines (AAs 210/211) within this putative NLS along with the adjacent arginine at AA 206 into alanines in both full length (ORF1M) and truncated (1-215 M) ORF1p constructs (Fig. 3a). Arginines were chosen for mutagenesis because they represent conserved amino acids of a monopartite NLS [63] and because mutation of all three of these arginines combined has previously been shown to abolish RNA binding of the full-length ORF1p [9]. Western blot analysis of the resulting proteins demonstrated that the mutant 1-215 M protein with mutations at aa positions 206/210/211 was detected predominantly in the cytoplasmic fraction compared to the wildtype 1–215 protein, which is detected in the nuclear fraction (Fig. 3 b, 1–215 versus 1-215 M). These findings are consistent with pNLS at AAs 201–215 function as a monopartite NLS in the context of the truncated 1–215 protein. In contrast, the same mutations in the full-length ORF1p had no effect on its subcellular localization (Fig. 3c). Mutations of other putative monopartite NLSs beginning at AAs 135 and 235, individually or in combination in the context of the full-length ORF1p, did not have any effect on the subcellular localization of the full-length protein (Additional file 2: Figure S2). It is important to note that mutation of the arginine at amino acid 235 has been previously shown to abolish RNA binding of ORF1p [9]. These findings demonstrate that truncated ORF1 proteins lacking RRM domain have different sequence requirements than the full-length ORF1p for their subcellular localization and that, although a monopartite pNLS at position 210–215 is sufficient for nuclear localization of a truncated ORF1 protein, sequences other than pNLSs must be involved in the nuclear localization of the full-length ORF1p. They also suggest potential redundancy in the sequence requirement for this process in the full-length ORF1p.

### Conserved lysines in the RRM and CTD play a role in the subcellular localization of the full-length ORF1p

In an attempt to determine the redundant amino acid sequences responsive for full-length ORF1p subcellular localization (beyond the above characterized pNLSs), we aligned the human and mouse ORF1p to identify conserved lysine residues. This approach is based on the fact that charged amino acids such as lysines and arginines are characteristic of non-canonical NLSs that typically rely on charge rather than sequence. Mouse and human ORF1p were chosen because they have been previously demonstrated to share structural and functional similarities [75, 76]. This analysis identified 14 lysines within the RRM and CTD that are conserved between mouse and human ORF1 proteins (Fig. 4a). These lysines identified in in the human ORF1p are conserved in the mouse ORF1p as either lysines or a biochemically similar amino acid [77]. We chose to mutate these residues because stretches of charged amino acids are typical of noncanonical NLSs [63–65]. Mutation of all 14 lysines into alanines dramatically shifted ORF1p localization from the nucleus to the cytoplasm (Fig. 4b). In order to assess the involvement of these lysine residues in the subcellular localization of ORF1p, we successively mutated 5 lysines within the RRM domain of a WT ORF1p into alanines to determine the effects of accumulated mutations (Fig. 4d). ORF1 proteins containing up to 5 mutated lysine residues (a group of mutants entitled “2-5KA”) exhibited little difference in subcellular localization relative to the wild type full-length ORF1p (Fig. 4c and d). However, 5KA ORF1 proteins containing additional mutations in either and/or both downstream mutant pairs (K272/274A, K295/300A) were detected predominantly in the cytoplasmic fraction and were expressed at significantly reduced levels compared to the wild type or 2-5KA ORF1 proteins (Fig. 4e). The fact that mutation of five lysines had no effect on the subcellular localization of the full-length ORF1p, but introduction of two independent sets of additional lysine mutations did, demonstrates redundancy in lysines required for ORF1p nuclear localization. This finding also suggests that there may be a critical number of charged amino acids needed for ORF1p localization to the nucleus.

Analysis of the residues crucial for ORF1p localization show high conservation. Alignment of the ORF1 RRM and CTD between L1PA1–8 and mouse L1 ORF1 show high conservation of the three arginine (Fig. 3) and 14 lysine (Fig. 4) residues shown to be crucial for truncated ORF1p and full-length ORF1p subcellular localization, respectively (Additional file 3: Figure S3). Two of the three arginines (human ORF1p aa 206 and 211) are conserved between L1PA1–8 and mouse L1 s and the third arginine (human ORF1p aa 210) is conserved among L1 subfamilies and appears as a lysine in the mouse ORF1 protein (Additional file 3: Figure S3). Additionally, seven out of 14 lysines are conserved within L1 subfamilies and seven out of 14 lysines are conserved in mouse L1 ORF1. All arginine and lysine substitutions are for biochemically similar amino acids [77]. Additionally, an analysis of ORF1 sequences from genomic full-length L1s [78] (primarily L1Hs subfamily) not surprisingly show high conservation of these residues among different L1Hs loci (Additional file 1).

### The lysine residues of RRM and CTD of the human ORF1p are involved in its interaction with flag-tagged KPNA2

To determine whether mutation of lysine residues abolishes previously reported ORF1p association with nuclear pore complex proteins, specifically KPNA2 [56], we performed co-immunoprecipitation experiments. We confirmed the previously reported interaction between flag-tagged ORF1p and untagged KPNA2 (Additional file 4: Figure S4A). Flag-tagged ORF1p associates with both endogenous and overexpressed KPNA2. As expected, flag-tagged ORF1p associates with endogenous KPNB1 as well (Additional file 4: Figure S4A). Co-immunoprecipitation analysis of overexpression of both flag-tagged ORF1p and wild type KPNB1 further confirms this association (Additional file 4: Figure S4B). Overexpression of KPNA2 increased the KPNA2 (but not KPNB1) signal in the co-IP fraction.

**Fig. 5:**
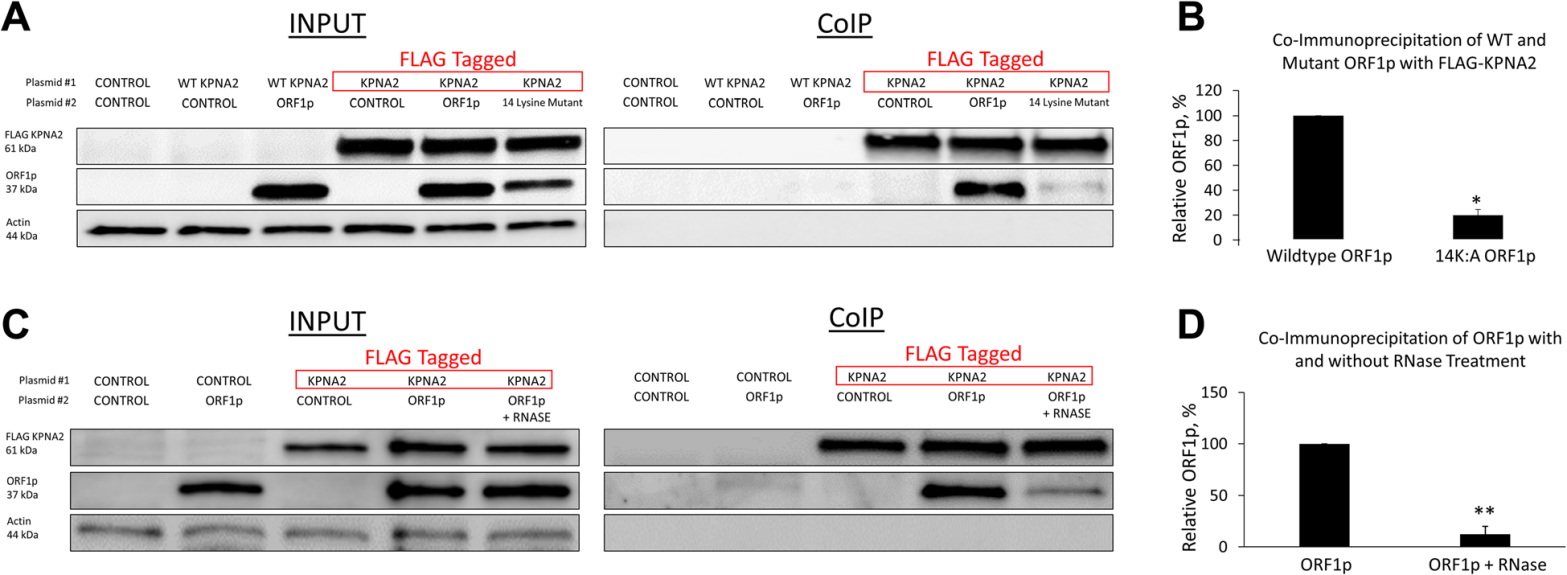
Interaction between ORF1p and KPNA2 is disrupted by either mutation of RRM and CTD lysines or RNase treatment. **a** HeLa cells were transiently co-transfected with plasmids expressing FLAG-tagged or untagged KPNA2 proteins and plasmids expressing wildtype or mutant human ORF1p. Co-Immunoprecipitation was performed with Anti-FLAG beads. Western blot analysis was performed using Anti-FLAG antibodies, hORF1 antibodies, and Actin loading control antibodies. Red boxes indicate FLAG-tagged proteins. Control indicates transfection with an empty plasmid. **b** Quantification of co-immunoprecipitation results shown as percentage of immunoprecipitated 14 K:A ORF1p as compared to Wildtype ORF1p. Error bars show standard deviation determined using data from three independent experiments (*, *p* < .05). **c** HeLa cells were transiently co-transfected with plasmids expressing FLAG-tagged KPNA2 proteins and plasmids expressing wildtype human ORF1p. Co-Immunoprecipitation was performed with Anti-FLAG beads. Western blot analysis was performed using Anti-FLAG antibodies, hORF1 antibodies, and Actin loading control antibodies. Red boxes indicate FLAG-tagged proteins. Control indicates transfection with an empty plasmid. **d** Quantification of co-immunoprecipitation results shown as percentage of immunoprecipitated ORF1p with RNase as compared to immunoprecipitated ORF1p without RNase. Error bars show standard deviation determined using data from three independent experiments (**, *p* < .01)

Co-immunoprecipitation using lysates from cells transiently transfected with plasmids containing sequences to express flag-tagged KPNA2 protein and untagged, wildtype or mutant, ORF1 proteins demonstrates that the loss of 14 lysines resulted in a 5-fold reduction in the levels of pulled ORF1 protein compared to the wild type ORF1p (Fig. 5a and b, ttest, *p* = 0.026).

### RNase treatment significantly decreases the extent of interaction between human ORF1p and flag-tagged KPNA2

In order to determine whether the KPNA2-ORF1p interaction is RNA-mediated, we performed a co-immunoprecipitation assay of transiently co-transfected FLAG-tagged KPNA2 and ORF1p followed by RNase treatment as previously described [56] (Fig. 5c). The RNase treatment greatly diminishes the KPNA2-ORF1p interaction, consistent with previous reports [56]. However, over 10% of these interactions persist even in the presence of RNase (Fig. 5d).

### Overexpression of both KPNA2 and KPNB1 increases L1 retrotransposition in HeLa cells

**Fig. 6:**
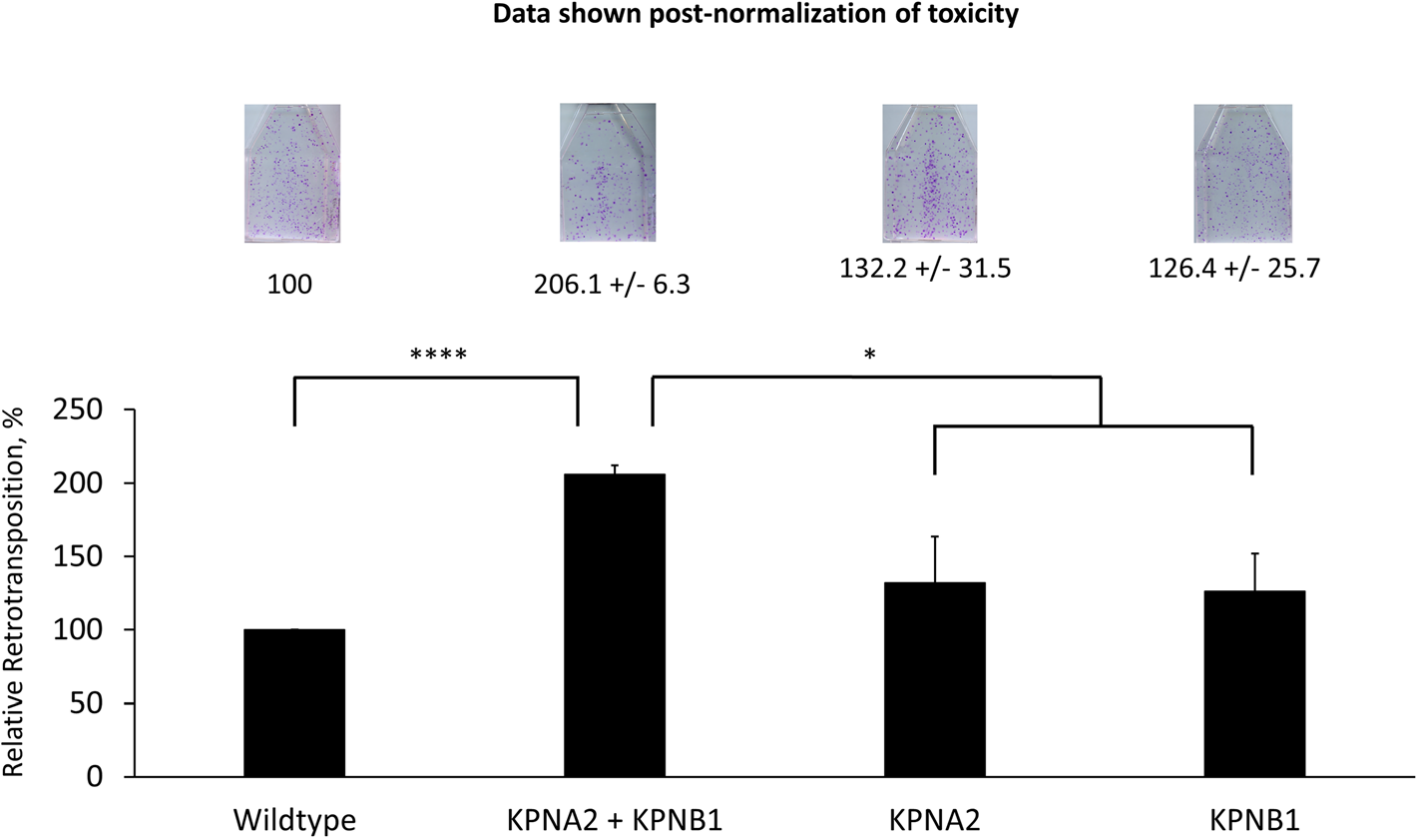
Transient overexpression of both KPNA2 and KPNB1 significantly increases L1 retrotransposition in HeLa cells. HeLa cells were transiently co-transfected with the L1Neo expression plasmid and plasmids expressing KPNA2 and/or KPNB1. All data normalized for toxicity that was determined by co-transfection of these expression plasmids with a plasmid expressing neomycin resistance gene (Additional file 5: Figure S5). Representative flasks are shown and the average number of colonies +/− standard deviation are indicated for each transfection condition. Error bars show standard deviation determined using data from three independent experiments (*, *p* < .05; ****, *p* < .0001)

After identifying specific amino acids in the human ORF1p that influenced ORF1p subcellular localization and are involved in association with KPNA2 and KPNB1, we sought to determine the effects of these proteins on L1 retrotransposition in HeLa cells as it was previously reported in HEK293T cells [56]. We used an engineered L1 reporter construct containing a reverse complimentary neomyocin-resistance reporter gene to detect de novo L1 insertion events [11]. In parallel, we transfected these test proteins in separate flasks to determine the effect of these proteins on cell viability (Additional file 5: Figure S5). These results are then adjusted to account for any resulting negative impact on cell viability. Though transfection of either KPNA2 or KPNB1 alone with the L1 reporter construct did not produce a notable change in L1 retrotransposition, co-transfection of both import proteins significantly increases L1 retrotransposition (Fig. 6, ttest, *p* value = <.0001). This is consistent with our co-IP result demonstrating that overexpression of KPNA2 alone did not increase the signal from endogenous KPNB1 (Additional file 4: Figure S4A). This is in accordance with their biological functions, as these proteins function as interacting partners for nuclear localization of the complex [62]. Though a greater than 2x increase in retrotransposition is seen between wildtype and co-transfection of both import proteins, these results are likely dulled by the high amounts of endogenous KPNA2 and KPNB1 in HeLa cells (Additional file 4: Figure S4A, CONTROL input lane).

Because a statistically significant decrease in L1 retrotransposition upon overexpression of KPNA2 in HEK293T cells was reported [56], but our studies detected an increase in retrotransposition in HeLa cells, we sought to compare mRNA expression of nuclear pore genes between the two cell lines. A comparison of RNA seq performed using RNA from the HeLa cell line used in the above retrotransposition assays [79] and publicly available RNA seq data for HEK293T cells shows a variation in the levels of KPNA2 and KPNB1 mRNA expression (FPKM) between these cell lines. The levels of these KPNA2 transcripts corresponded to the reported KPNA2 protein levels in these cell lines (https://www.novusbio.com/PDFs3/NBP2-52501.pdf). This analysis also determined that other members of KPNA family are expressed in both cell lines suggesting a possibility of a complex, potentially tissue-specific, interplay in their involvement in nuclear transport. Based on our RNA-seq analysis, we were unable to make informative conclusions as to whether the composition of expressed KPNA genes is directly responsible for the observed differences in L1 retrotransposition upon KPNA2 and KPNB1 overexpression. As many differences between cell lines exists, it is possible that the observed effect on L1 retrotransposition in each cell line is not due to the increase in the direct interaction between ORF1p with the nuclear pore proteins, but rather because of a secondary effect of KPNA2/KPNB1 overexpression on nuclear transport of host proteins that may facilitate or suppress L1 mobilization.

### ORF1p interacts with other members of the KPNA family of proteins

**Fig. 7:**
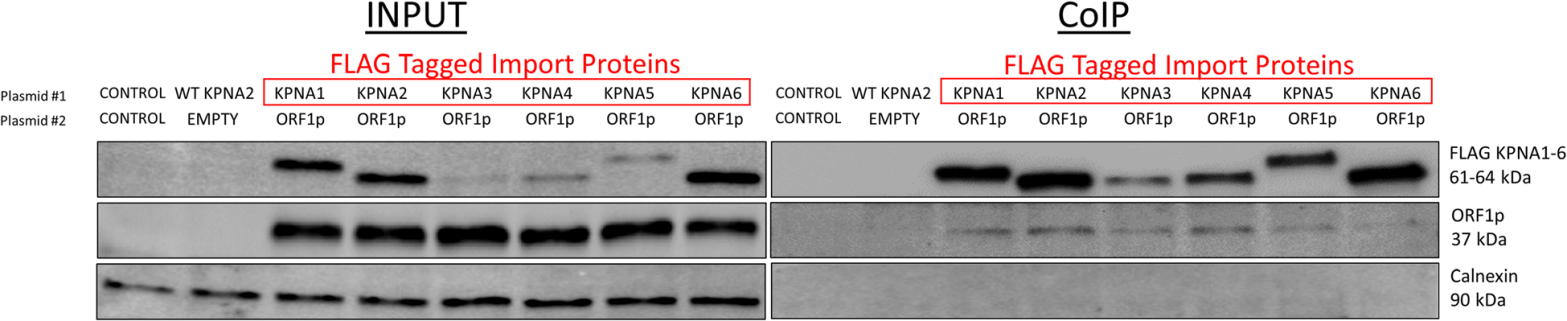
ORF1p interacts with members of the KPNA family of importin proteins. HeLa cells were transiently co-transfected with a plasmid expressing one of the FLAG-tagged KPNA1–6 proteins and a plasmid expressing untagged human ORF1p. Co-Immunoprecipitation was performed with Anti-FLAG beads. Western blot analysis was performed using Anti-FLAG antibodies, hORF1 antibodies, and Calnexin loading control antibodies. Red boxes indicate FLAG-tagged proteins. Control indicates transfection with an empty plasmid

Analysis of RNA-Seq datasets generated using HeLa or HEK 293 T cells determined that these cells support expression of KPNA1–6 mRNA (Additional file 6: Table S1). To investigate whether other KPNA family members associate with human ORF1p (Fig. 6), we performed co-immunoprecipitation using cells co-transfected with a plasmid expressing one of the flag-tagged KPNA1–6 proteins and a plasmid expressing untagged ORF1p. Although the flag-tagged KPNA proteins were expressed at different levels, they all associated with the human ORF1p to varying degrees (Fig. 7).

## Discussion

L1 retrotransposition requires L1 mRNA and its associated proteins to function in both the nuclear and cytoplasmic cellular compartments [6]. The retrotransposition process begins with L1 mRNA transcription in the nucleus and continues with its translation in the cytoplasm [80, 81]. The two resulting proteins, ORF1p and ORF2p, associate with their parental mRNA (termed cis preference) in the cytoplasm, forming the L1 RNP [11, 14, 15], which is considered to be a retrotransposition intermediate. The L1 RNP gains access to DNA through a poorly understood mechanism and undergoes TPRT, a process of reverse transcribing L1 mRNA and creating a novel L1 insert in the genome [23, 24]. We have previously reported the existence of L1 loci in the human genome that contain stop codons in their ORF1 sequence. We demonstrated that full-length ORF1p as well as several of these truncated proteins suppress retrotransposition of engineered human L1. These findings support that in addition to understanding of ORF1p contribution to the subcellular localization of L1 RNPs, there is also a need to understand subcellular trafficking of non-specific ORF1p complexes.

Understanding the function of L1 proteins in different cellular compartments is complicated by the fact that endogenous L1 expression is very low, thus L1 proteins are rarely detected in normal cells [37, 82]. However, a recent publication reported detection of endogenous L1 ORF1p in the nucleus [44]. An increase in expression of L1 ORF1 protein is detected in some human cell lines, germ cells, and in a variety of tumor samples from cancer patients [28, 33–37]. Specifically, it has been reported that nuclear ORF1p signal correlates with poor prognosis in breast cancer patients [38, 39]. These findings support that discovering mechanisms involved in trafficking of L1 ORF1p inside host cells may be useful for better understanding its contribution to the steps of the L1 life cycle that take place in different cellular compartments. Studies using western blot analysis and immunohistochemistry have shown that ORF1p localization, both endogenous and overexpressed, could vary depending on cell lines and tumor types, implying a possibility that these differences may be important for L1 progression through its amplification steps [40–44]. Tagged ORF1p has been shown to vary in its localization patterns from the untagged ORF1p [40, 41, 69] and several tagged ORF1p versions significantly reduced or even abolished L1 retrotransposition in human cell lines [22]. Results collected using immunohistochemistry and transient transfections of C-terminally GFP-tagged ORF1p (ORF1p-GFP) in 143Btk- and 293 T cells, show that a GFP-tagged full length ORF1p is largely cytoplasmic. Truncation analysis of ORF1p-GFP determined that the N-terminal third of the human ORF1p (consisting of the NTD and part of the CCD) is important for cytoplasmic retention of the protein. The same analysis indicated that a sequence downstream of the N-terminal third of ORF1p may be important for nuclear localization of the ORF1p-GFP protein [69].

With the development of antibodies that directly detect ORF1p, it is now possible to study subcellular localization of untagged ORF1p [39–41, 44, 70, 72]. We and others have reported that untagged human and mouse ORF1p transiently transfected in human or mouse cells are detected in both the cytoplasmic and nuclear fractions [16, 40, 42, 44]. Additionally, we have reported a difference in the subcellular localization of the full-length and some C-terminally truncated ORF1 proteins [41], suggesting that it is possible to identify sequences within the ORF1 protein that may be dictating its subcellular localization without any potential interference from a tag. Interestingly, untagged truncated ORF1p protein containing only the NTD, the RRM, or the CTD were not stable in our system (Fig. 1), suggesting that the addition of a tag in previous studies may stabilize certain ORF1p fragments. Using a truncation approach similar to the previously reported one [69], combined with systematic mutation analysis of putative NLSs predicted in the human ORF1p sequence, we determined that charged amino acids in the RRM and CTD of the human ORF1p are required for its detection in the nuclear fraction of HeLa cells. In addition, our results show a substantial difference in the amino acid requirements for nuclear localization of the full length ORF1p and the truncated 1–215 proteins. The wild type full-length ORF1p and the 1–215 proteins are predominantly nuclear (Figs. 3 and 4). The fact that the two arginine residues at positions 261 and 262 that are essential for RNA binding are missing in the 1–215 protein supports that its subcellular localization is not dependent on RNA-binding. The difference in the behavior of mutant forms of these two proteins is exemplified by the cytoplasmic localization of the 1-215 M protein and the nuclear localization of the ORF1M protein (Fig. 3b vs c), both containing the triple arginine mutation within the RRMThis difference could be explained by the redundancy of the sequences contributing to the nuclear localization of the full-length construct, which is most likely present in the ORF1p portion that is absent within the 1–215 construct (Fig. 3a). This portion of the protein contains multiple charged amino acids that could be important for nuclear localization. It is also possible that structural changes due to truncation may be a contributing factor to the differences in subcellular localization, as the truncated 1–215 construct is unlikely to bind RNA.

This observation, combined with the fact that mutations introduced to disrupt putative canonical NLSs identified in the ORF1p sequence did not result in any changes in ORF1p subcellular localization, led us to consider a possibility that noncanonical NLSs within the RRM and/or CTD may be involved. A noncanonical NLS depends on regional charge and 3D structure as opposed to amino acid sequence specificity. While canonical NLS sites contain a rigid amino acid sequence, a nonspecific cluster of basic residues that comprise a noncanonical NLS can also bind to import proteins [63–65].

Guided by this information, we determined that mutation of 14 lysines present in the RRM and CTD is sufficient to eliminate detection of ORF1p in the nuclear fraction of HeLa cells. Site-directed mutagenesis of five of these amino acids (K227, K229, K237, K243, and K245) in the RRM domain, individually or in combination, determined that mutation of this initial cluster of lysines does not substantially affect subcellular localization of human ORF1p. Mutation of these 5 lysines and two additional non-identical downstream lysines severely inhibits nuclear localization of ORF1p (Fig. 4), suggesting there may be a critical number of these charged amino acids that confer ORF1p presence in the nuclear fraction. Additionally, as discussed above, elimination of three arginines in the 1–125 construct, R206, R210, and R211, led to detection of the truncated, but not the full-length, ORF1p predominantly in the cytoplasmic fraction of Hela cells (Fig. 3b). Interestingly, our previous work demonstrated that cytoplasmic variants of truncated ORF1p could be shuttled into the nucleus by the wild type full-length ORF1p [41], suggesting that they are incorporated into the ORF1p trimers or there is some form of cooperation between trimers composed of the full-length ORF1p and those formed by the truncated proteins that facilitates their excess to the nucleus.

Identification of sequences within the human ORF1p that alter its subcellular localization and that are consistent with the features of a non-canonical NLS supports is consistent with the reported interaction between ORF1p and nuclear pore proteins. While ORF1p interaction with KPNA2 has been previously shown with mass spectrometry and co-immunoprecipitation [56], our result has identified ORF1p sequence requirements for this interaction. ORF1p containing mutations of 14 lysines and exhibits cytoplasmic localization also loses its interaction with import proteins KPNA and KPNB as demonstrated by co-IP analysis (Fig. 5). We also demonstrate that co-transfection of both KPNA2 and KPNB1 significantly increases L1 retrotransposition in Hela cells, though it is unclear as to whether this effect is due exclusively to their impact on ORF1p or other cellular factors that may influence L1 retrotransposition (Fig. 6). Our co-IP approach also demonstrates an association of ORF1p with other KPNA family proteins (Fig. 7) and RNA-seq analysis determined that different members of the KPNA family are co-expressed, suggesting that there may be redundancy or a cell type-specificity in the ORF1p interaction with the nuclear pore complexes.

Previous immunoprecipitation studies have shown a significant decrease in interaction between flag-tagged ORF1p and KPNA2 after RNase treatment, indicating a role for nucleic acid binding for the association between ORF1p and the KPNA family of import proteins [56]. The three conserved arginines within the RRM (AAs 206/210/211) that were mutated in our study have been previously shown to abolish RNA binding of the full-length ORF1p, but their mutation does not affect subcellular localization of the protein (Fig. 3C). Mutation of the arginine at position 235 within the RRM has also been previously shown to abolish RNA binding [9], however, a full-length ORF1p containing this mutation, as well as mutation of amino acids 236–238, behaves similar to the wild type protein regarding its subcellular localization (Additional file 2: Figure S2B). Additionally, many amino acids essential for ORF1p RNA binding are removed in the truncated 1–215 construct, yet this truncated protein is detected in the nucleus. Combined, these findings support that RNA binding ability of the human ORF1p may not be required for its nuclear localization, but rather suggest the possibility that both RNA bound and unbound ORF1p can interact with the nuclear pore proteins [9, 50, 83, 84].

To test this possibility experimentally, we used RNase treatment that was previously reported to disrupt ORF1p interactions with some cellular factors, including KPNA2. The significant loss of KPNA2 ORF1p interaction upon RNase treatment (Fig. 5c and d) is in agreement with the previous report [56]. This result supports a possibility that the interaction between these two proteins is indirect and completely RNA-dependent with an incomplete RNA digest being an explanation for the remaining ORF1p signal. This is, however, unlikely as our method of RNase treatment has been previously shown to completely diminish RNA-mediated interactions [56]. Alternatively, the abundance of ORF1p within ORF1p:RNA complexes with only some ORF1p molecules interacting with KPNA2 at any given time may explain the observed significant loss of KPNA2-ORF1p interaction. If a small fraction (10% based on Fig. 5c and d) of ORF1p trimers mediate a direct KPNA2-ORF1p interaction at any given time, it is likely that the entire ORF1p-RNA-containing complex is then pulled down with KPNA2 in our experiment. Based on this possibility, RNase treatment would decrease the ORF1p signal by leaving only trimers directly bound to KPNA2. Therefore, it is possible that it is these trimers that are bound directly to KPNA2 that we detect in the RNase treated FLAG-tagged KPNA2 pulldown.

High conservation of the residues mediating ORF1p localization is seen in alignments of human/mouse interspecies ORF1 sequences, L1PA1–8 subfamilies, and 134 L1Hs loci sequences (Fig. 5, Additional file 3: Figure S3, Additional file 7). This suggests a possible selective pressure in conserving the three arginine (Fig. 3) and 14 lysine (Fig. 4) residues shown to be crucial for truncated ORF1p or full-length ORF1p subcellular localization, respectively. The high conservation seen throughout the RRM and CTD (Fig. 4a) could be due to the importance of these domains for both previously identified RNA binding [9] and herein reported nuclear localization. Our finding that as many as seven lysine residues need to be mutated in order to affect full-length ORF1p subcellular localization, while mutations of only two or three charged amino acids abolish ORF1p RNA binding support that there may be a positive selection for these lysines due to their involvement functionally or structurally in nuclear localization of ORF1p present either in the L1 RNPs or non-specific ORF1p complexes.

## Conclusions

The prediction of NLS sequences suggested that specific sequences within ORF1p could be responsible for its subcellular localization by interacting with nuclear binding proteins. The authors have identified specific charged amino acids in the C-terminal end of ORF1p that mediate this property (functionally or structurally), show high conservation between human and mouse ORF1p, and interact with KPNA2 protein, which is a key component of the cellular nuclear pore complex. Mutated ORF1p with cytoplasmic localization constructs lose their interaction with nuclear pore proteins, a finding consistent with these charged residues functioning as a noncanonical NLS or introduce structural changes that prevent proper protein-protein interactions. Mutation and deletion analyses demonstrate that the full length ORF1p and truncated ORF1p have different amino acid requirements for subcellular localization, which may have differential impact on non-specific ORF1p complexes. An increase in L1 retrotransposition upon overexpression of KPNA2 and KPNB1 in HeLa cells combined with our finding of a novel association between ORF1p and other KPNA family proteins suggest that some redundancy in ORF1p nuclear pore interactions may exist in a tissues- or cell type-specific manner.

## Materials and methods

### Generation of ORF1-containing plasmids

The 1–54 (54co), 1–132 (132co), 1–157 (157co), 1–255 (255co) and CCD (53-157co) constructs were previously described [41], Lysine mutant (ORF1 ub) construct was previously described [72]. These sequences were subcloned into the pBud plasmid (Invitrogen) using HindIII and BamHI restriction endonucleases.

### Cell culture

HeLa cells were cultured as previously described [85].

### Transfection

HeLa cells were seeded at 2 × 10^6^ cells per T75 flask and transfected 16–18 h later with 3 μg of expression plasmids containing codon-optimized ORF1 sequence. Plus reagent (6 μl) (Invitrogen) and lipofectamine (12 μl) (Invitrogen) were used in the transfection reaction in serum-free media. Serum-free media was replaced with serum-containing media 3 h after transfection and cells were harvested 24 h later.

### Protein harvest for western blot analysis

Cells were washed with 1X PBS (137 mM NaCl (Sigma S9888), 2.7 nM KCl (Sigma P4505), 10 mM Na2HPO4 (Sigma S3264) and 2 mM KH2PO4 (Sigma P9791), pH = 7.4) and harvested using 500 μl of TLB (50 mM Tris, 150 mM NaCl, 10 mM EDTA, TritonX-100 0.5% *v*/v, Halt Protease inhibitor 10 μl/mL, phosphatase inhibitors 2 and 3 (Sigma), pH = 7.2) buffer per T75 flask. The samples were centrifuged at 21130×g for 15 min at 4-degrees Celsius. The supernatant was transferred to a new microcentrifuge tube (this is the cytoplasmic fraction). The remaining nuclear pellet in the microcentrifuge was gently washed three times with 200ul of TLB buffer. The nuclear pellets were lysed with TLB SDS, (50 mM Tris, 150 mM NaCl, 10 mM EDTA, 0.5% sodium dodecyl sulfate, TritionX 0.5%, pH = 7.2) and the nuclear lysate samples were sonicated three times for 10 s at 12 watts RMS using a 3 mm wide Qsonica Microson homogenizer with Microson ultrasonic cell disruptor XL2000 (Microson). Samples were centrifuged at 21130×g for 15 min at 4-degrees Celsius. The resulting supernatant was transferred to a new microcentrifuge tube. This supernatant is the nuclear fraction. The protein concentration for each sample was determined using 595 nm wavelength OD values against a Bovine Serum Albumin (BSA) standard.

### Western blot analysis

15-40 μg of protein for each sample was combined with 2x Laemmli buffer to obtain the final concentration of 1X, 1.6 μl β-mercaptoethanol and heated at 100-degrees Celsius for 5 min prior to loading. Equivalent amounts of protein were loaded in the nuclear and cytoplasmic fractions when applicable. Samples were fractionated on a Bis-Tris 4–12% Midi gel (Invitrogen) and transferred to a nitrocellulose membrane (iBlot2 system: Invitrogen). The membrane was first incubated for 1 h in the 5% milk/PBS-Tween buffer (0.1% *v*/v Tween 20 (Sigma P2287), 137 mM NaCl (Sigma S9888), 2.7 nM KCl (Sigma P4505), 10 mM Na2HPO4 (Sigma S3264) and 2 mM KH2PO4 (Sigma P9791), pH = 7.4), and then overnight at 4-degrees Celsius with 1:5000 dilution of hORF1 (custom polyclonal rabbit antibody: TGNSKTQSASPPPK) antibody or 1:5000 dilution of hORF1 201 (custom polyclonal rabbit antibody: QRTPQRYSSRRATP) antibody or 1:15000 T7-tag (Cell Signaling; D9E1X) or 1:1000 eIF3n antibody (Santa Cruz; sc-16,377) or 1:10000 FLAG antibody (Cell Signaling; #2368) or 1:1000 KPNB antibody (Cell Signaling; E1F1E) antibody in 3% milk/PBS-Tween buffer. Following the overnight incubation, the membranes were washed for 5 min 3 times with PBS-Tween buffer, and incubated at room temperature for 1 h with 1:5000 dilution of horseradish peroxidase-conjugated secondary antibodies (HRP-donkey anti-rabbit (Santa Cruz: sc2317) or HRP-goat anti-mouse (Santa Cruz: sc2031)) in 3% milk/PBS-Tween buffer. The membranes were washed one time for 15 min with PBS-Tween buffer then twice for 5 min with PBS-Tween buffer. The development was done using a 5-min incubation with a Bio-Rad Clarity Kit (Bio-Rad 1,705,061). The signal was detected using a Chemi-Doc XRS+ Molecular Imager (Bio-Rad). GAPDH antibodies (Santa Cruz: sc-25,778, 1:5000 dilution) and Lamin A/C (Santa Cruz 7293, 1:1000 dilution) were used as a fractionation and equal loading controls.

### Co-immunoprecipitation

Adapted from Sokolowski et al. [41] Transfection for Co-immunoprecipitation: 2 × 10^6^ HeLa cells per T75 flask were seeded 16–18 h prior to transfection. The cells were co-transfected with 3 μg of either a control (pBud) or a test plasmid and 3 μg of either control (pBud) or another test plasmid using 12 μl of Plus reagent in the total volume of 200 μl of serum-free media. After 10 min of incubation at room temperature, 24 μl of Lipofectamine mixed with 76 μl of serum-free DMEM/High Glucose media was added to the reaction. The transfection cocktail was incubated for 15 min at room temperature and transferred into individual flasks containing 6 mL of serum-free DMEM/High Glucose media. 3 h post transfection, the media was replaced with 8 mL of serum containing media. Cells were harvested approximately 24 h post transfection. Protein harvest for Co-immunoprecipitation: Cells were washed with 1X PBS (137 mM NaCl (Sigma S9888), 2.7 nM KCl (Sigma P4505), 10 mM Na_2_HPO_4_ (Sigma S3264) and 2 mM KH_2_PO_4_ (Sigma P9791), pH = 7.4) and harvested using 500 μl of TLB lysis buffer (50 mM Tris, 150 mM NaCl, 10 mM EDTA, TritonX-100 0.5% *v*/v, Halt Protease inhibitor 10 μl/mL, phosphatase inhibitors 2 and 3 (Sigma), pH = 7.2) per T75 flask. Samples were centrifuged at 18407×g for 15 min at 4-degrees Celsius. The resulting supernatant was transferred to a new microcentrifuge tube (input). When applicable, input was split in two tubes, one of which contained 2 μl of RNaseA (ThermoFisher EN0531). The following steps were carried out at 4-degree Celsius. 40 μl of flag resin (Anti-Flag M2 Affinity Gel Sigma A2220) was centrifuged at 8200×g for 30 s, and 1 min incubation prior to removing the supernatant of the resin. The resin was washed twice using 500 μl of TBS (50 mM Tris HCl,150 mM NaCl, 1 mM EDTA, 1% Triton X-100 v/v, pH = 7.4). 200 μl of the protein sample was combined with 800 μl of TBS buffer incubated with the prepared flag resin overnight at 4-degrees Celsius on a revolving platform. The following day, the mixture was centrifuged at 8200×g for 30 s. The supernatant was removed and the resin was washed three times with 500 μl of TBS. After the washes, the remaining protein was eluted by incubation at 100-degrees Celsius for 3 min in 20 μl of 2X sample buffer (125 mM Tris HCl, 4% SDS, 20% (v/v) glycerol, 0.004% bromophenol blue, pH = 6.8). The eluted samples were centrifuged for 30 s at 8200×g and the supernatant was used for western blot analysis (the co-IP fraction).

### L1 Retrotransposition assay

Adapted from Sokolowski et al. [41] 5 × 10^5^ HeLa cells per T75 flask were seeded 16–18 h prior to transfection. The cells were co-transfected with 0.1 μg of L1Neo plasmid and 0.2 μg of KPNA2 and/or KPNB1 plasmid and control plasmid (pBud) up to 0.5μg total using 4 μl of Plus reagent in the total volume of 200 μl of serum free media. In parallel, results were normalized to toxicity of test plasmids by setting up identical transfection replacing 0.1 μg of L1Neo plasmid with 0.1 μg of control plasmid. After 10 min of incubation at room temperature, 8 μl of Lipofectamine mixed with 92 μl of serum-free DMEM/High Glucose media was added to the reaction. The transfection cocktail was incubated for 15 min at room temperature and transferred into individual flasks containing 6 mL of serum-free DMEM/High Glucose media. 3 h post transfection, the media was replaced with 10 mL of serum containing media. 400 μg/mL of G418 (Geneticin, Invitrogen: 10131–027) was administered 24 h post transfection and maintained for up to 14 days with media changes every 2–3 days. The flasks were stained with 3 mL of crystal violet staining solution (0.2% crystal violet (Sigma C6158), 5% acetic acid (Fisher Scientific A38–212), 2.5% isopropanol (Fisher Scientific BP2632–4)) per flask.

### RNA-Seq data generation

HeLa cells RNA seq data comes from data set as previously described [79]. HEK293T cells RNA seq data comes from data set publically available through NCBI SRA (SRR1182596). RSEM analysis was used to calculate FPKM expression values.

### ORF1p amino acid alignment

Amino acid sequence alignment performed using MegAlign software (DNASTAR v. 10.0.1). Sequences aligned using either Lipman-Pearson method (Fig. 4 and Additional file 3: Figure S3) or clustal W method (Additional file 7) relative to the human ORF1p L1PA1 sequence.

## Additional files

Additional file 1: **Figure S1.** Quantification of Fig. 1. Quantification of Fig. 1C. Error bars show standard deviation determined using data from 2 independent experiments (*, *p* < .05). (TIF 83 kb)

Additional file 2: **Figure S2.** Mutation of the putative monopartite nuclear localization signal in ORF1. **A**.ORF1 domains are indicated as an N-terminal domain (N), a coiled-coil domain (CCD), and RNA recognition motif (RRM) and a C-terminal domain (CTD). Positions of domains shown are approximate.The antibody symbol (name above) denotes approximate location of the antibody on the ORF1p. Putative bipartite nuclear localization signal (Bipartite) is shown in green and putative monopartite nuclear localization signal (monopartite) is shown in brown. The red “X” (X) denotes the mutation of the listed amino acid positions, on the left, into alanine residues. The expected molecular weight of each construct is listed to the right. **B.** Western blot analysis of full-length human ORF1 transiently transfected in HeLa cells. Proteins are separated into nuclear (N) and cytoplasmic (C) fractions. Human ORF1 protein was detected with human-specific ORF1 polyclonal antibodies (hORF1). Calregulin (cytoplasmic marker) and Lamin A/C (nuclear marker) are used as loading and cell fractionation controls. Positions of molecular markers are indicated on the right in kDa. Control indicates cells transfected with empty plasmid. (TIF 219 kb)

Additional file 3: **Figure S3.** Conservation of residues important for ORF1p localization between mouse and human L1 subfamilies. Schematic of part of the RNA recognition motif (RRM) and C-terminal domain (CTD) of ORF1. Positions of domains shown are approximate. Human ORF1p L1PA1–8 (top) is aligned to mouse ORF1p (bottom) using Lipman-Pearson method. Yellow stars denote the amino acid position of the arginine residues and purple stars denote the amino acid position of the lysine residues that were mutated to alanine residues in the human ORF1. Boxed residue indicate amino acids different from L1PA1 ORF1. (TIF 971 kb)

Additional file 4: **Figure S4.** Flag-tagged ORF1p forms import complex with KPNA2 and KPNB1 import proteins. ***A.*** *HeLa* cells were transiently co-transfected with plasmids containing FLAG-ORF1p and/or KPNA2. Co-Immunoprecipitation was performed with Anti-FLAG beads. Western blot analysis was performed using KPNA2 antibodies, KPNB1 antibodies, Anti-FLAG antibodies, and GAPDH loading control antibodies. Red boxes indicate FLAG-tagged proteins. Control indicates transfection with an empty plasmid. **B.** HeLa cells were transiently co-transfected with plasmids containing FLAG-ORF1p and/or KPNA2. Co-Immunoprecipitation was performed with Anti-FLAG beads. Western blot analysis was performed using KPNB1 antibodies, Anti-FLAG antibodies, and GAPDH loading control antibodies. Red boxes indicate FLAG-tagged proteins. Control indicates transfection with an empty plasmid. Three micrograms of each plasmid was transfected unless otherwise indicated by (#). (TIF 512 kb)

Additional file 5: **Figure S5.** Toxicity assay of KPNA2 and/or KPNB1 in HeLa cells. Toxicity determined by co-transfection of KPNA2 and/or KPNB1 with a plasmid expressing neomycin resistance gene. Average number of colonies are indicated for each transfection condition. Error bars show standard deviation determined using data from 3 independent experiments (**, *p* < .01; ****, *p* < .0001). (TIF 55 kb)

Additional file 6: **Table S1.** Expression profile of import genes in HEK293T cells and HeLa cells. Expression profiles for HeLa cells were determined using RNA-seq dataset79. Expression profiles for HEK293T cells were determined using RNA-seq data publically available through NCBI SRA (SRR1182596). Data calculated as fragments per kilobase of transcript per million mapped reads (FPKM). (TIF 140 kb)

Additional file 7: Alignment of ORF1 sequences from genomic L1s. Alignment performed using clustal W method relative to the human ORF1p L1PA1 sequence. (PDF 75 kb)

## Abbreviations

CCD: Coiled coil domain
CTD: C-terminal domain
L1 or LINE- 1: Long interspersed element 1
NLS: Nuclear localization signal
NTD: N-terminal domain
ORF: Open reading frame
RNP: Ribonucleoprotein
RRM: RNA recognition motif
TPRT: Target-primed reverse transcription
