# Supplementary figures and images for: Identification of charged amino acids required for nuclear localization of human L1 ORF1 protein
Source: Mob DNA. 2019 May 6;10:20. doi: 10.1186/s13100-019-0159-2 (PMC6501352; doi:10.1186/s13100-019-0159-2)

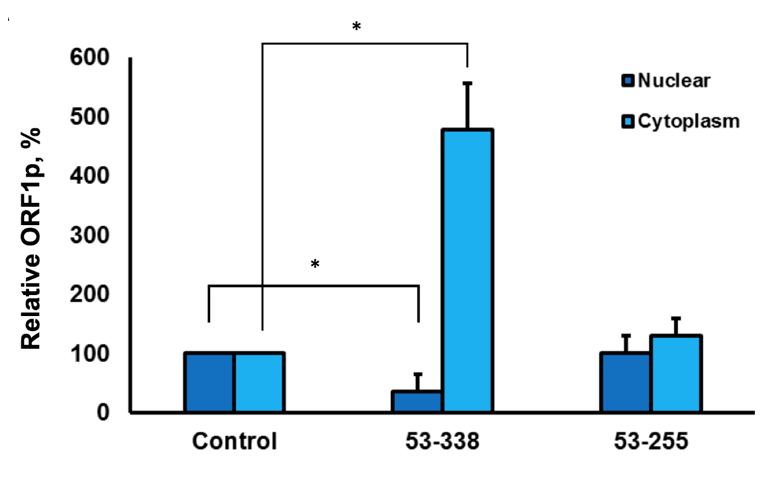

Supplement: Supplementary file 1 — Figure S1. Quantification of Fig. 1. Quantification of Fig. 1C. Error bars show standard deviation determined using data from 2 independent experiments (*, p < .05). (TIF 83 kb) [file 13100_2019_159_MOESM1_ESM.tif]

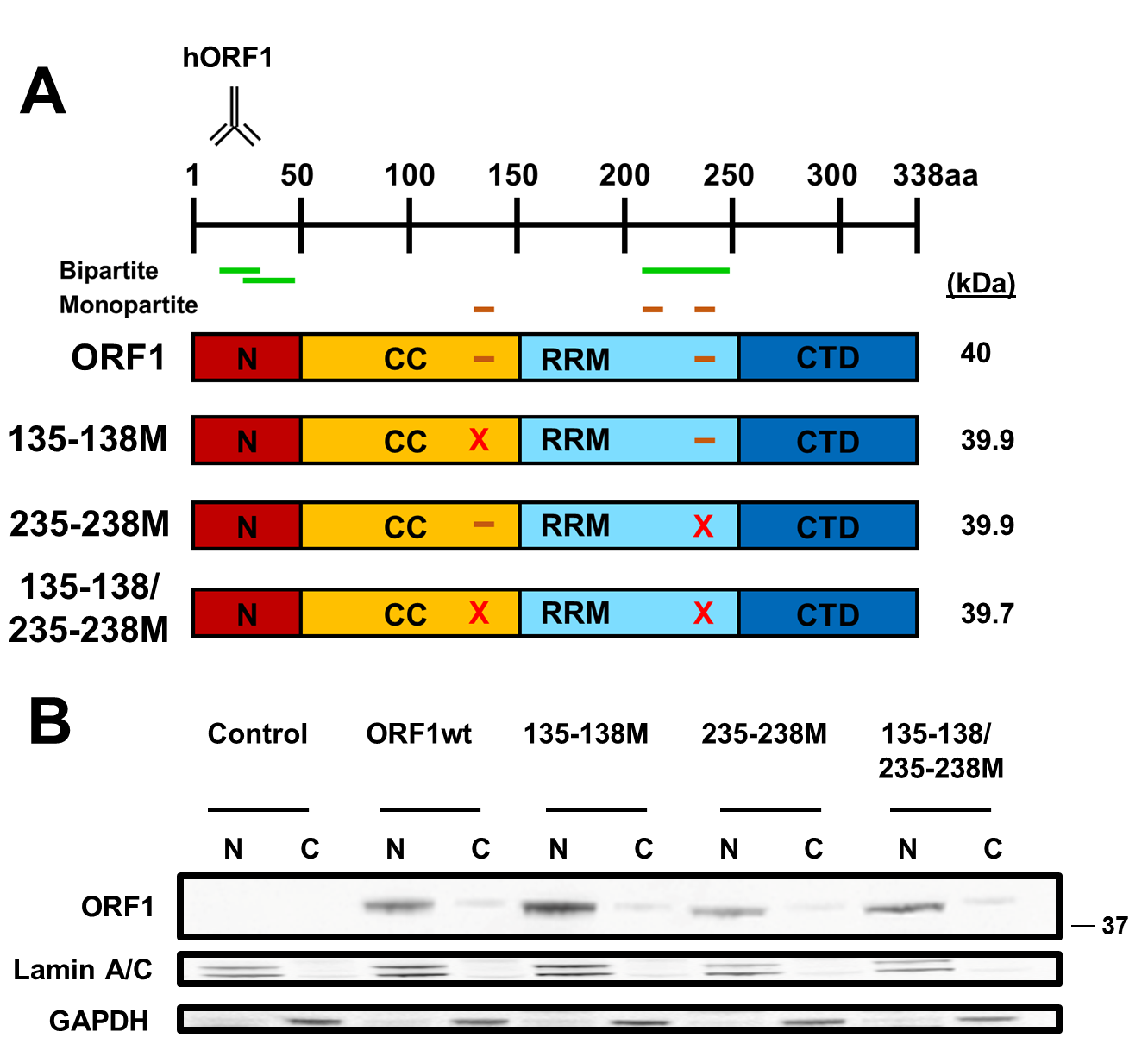

Supplement: Supplementary file 2 — Figure S2. Mutation of the putative monopartite nuclear localization signal in ORF1. A.ORF1 domains are indicated as an N-terminal domain (N), a coiled-coil domain (CCD), and RNA recognition motif (RRM) and a C-terminal domain (CTD). Positions of domains shown are approximate.The antibody symbol (name above) denotes approximate location of the antibody on the ORF1p. Putative bipartite nuclear localization signal (Bipartite) is shown in green and putative monopartite nuclear localization signal (monopartite) is shown in brown. The red “X” (X) denotes the mutation of the listed amino acid positions, on the left, into alanine residues. The expected molecular weight of each construct is listed to the right. B. Western blot analysis of full-length human ORF1 transiently transfected in HeLa cells. Proteins are separated into nuclear (N) and cytoplasmic (C) fractions. Human ORF1 protein was detected with human-specific ORF1 polyclonal antibodies (hORF1). Calregulin (cytoplasmic marker) and Lamin A/C (nuclear marker) are used as loading and cell fractionation controls. Positions of molecular markers are indicated on the right in kDa. Control indicates cells transfected with empty plasmid. (TIF 219 kb) [file 13100_2019_159_MOESM2_ESM.tif]

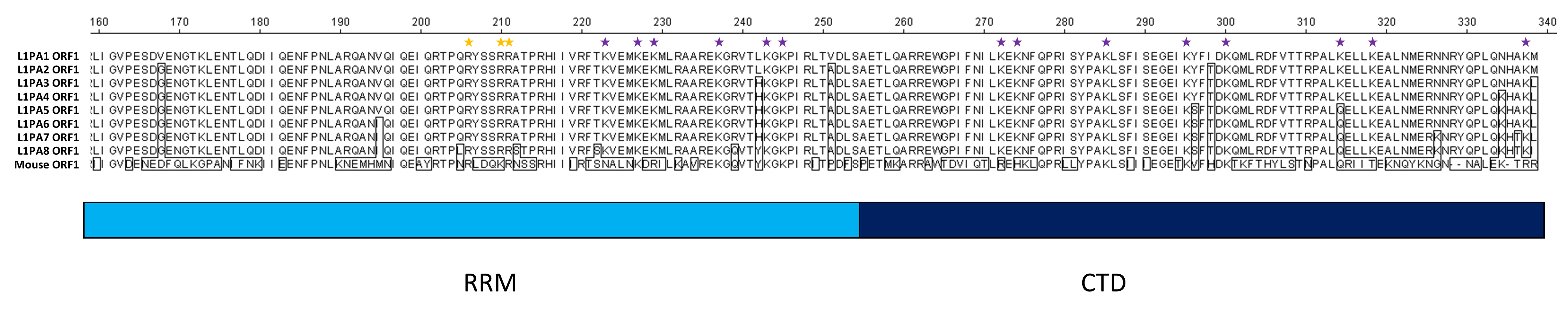

Supplement: Supplementary file 3 — Figure S3. Conservation of residues important for ORF1p localization between mouse and human L1 subfamilies. Schematic of part of the RNA recognition motif (RRM) and C-terminal domain (CTD) of ORF1. Positions of domains shown are approximate. Human ORF1p L1PA1–8 (top) is aligned to mouse ORF1p (bottom) using Lipman-Pearson method. Yellow stars denote the amino acid position of the arginine residues and purple stars denote the amino acid position of the lysine residues that were mutated to alanine residues in the human ORF1. Boxed residue indicate amino acids different from L1PA1 ORF1. (TIF 971 kb) [file 13100_2019_159_MOESM3_ESM.tif]

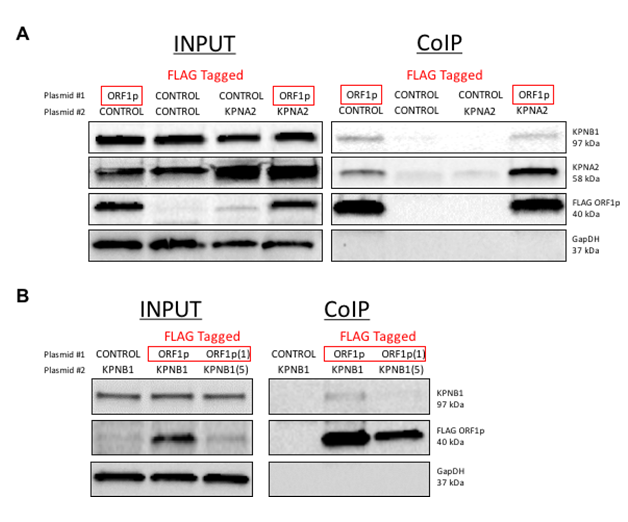

Supplement: Supplementary file 4 — Figure S4. Flag-tagged ORF1p forms import complex with KPNA2 and KPNB1 import proteins. A. HeLa cells were transiently co-transfected with plasmids containing FLAG-ORF1p and/or KPNA2. Co-Immunoprecipitation was performed with Anti-FLAG beads. Western blot analysis was performed using KPNA2 antibodies, KPNB1 antibodies, Anti-FLAG antibodies, and GAPDH loading control antibodies. Red boxes indicate FLAG-tagged proteins. Control indicates transfection with an empty plasmid. B. HeLa cells were transiently co-transfected with plasmids containing FLAG-ORF1p and/or KPNA2. Co-Immunoprecipitation was performed with Anti-FLAG beads. Western blot analysis was performed using KPNB1 antibodies, Anti-FLAG antibodies, and GAPDH loading control antibodies. Red boxes indicate FLAG-tagged proteins. Control indicates transfection with an empty plasmid. Three micrograms of each plasmid was transfected unless otherwise indicated by (#). (TIF 512 kb) [file 13100_2019_159_MOESM4_ESM.tif]

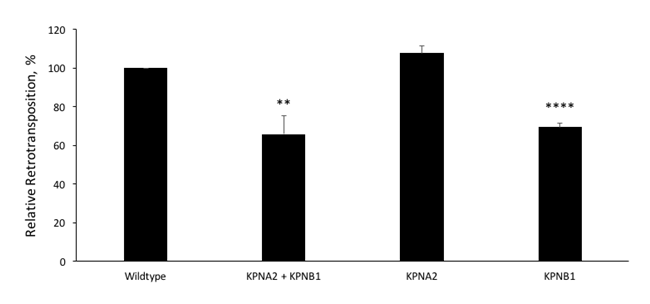

Supplement: Supplementary file 5 — Figure S5. Toxicity assay of KPNA2 and/or KPNB1 in HeLa cells. Toxicity determined by co-transfection of KPNA2 and/or KPNB1 with a plasmid expressing neomycin resistance gene. Average number of colonies are indicated for each transfection condition. Error bars show standard deviation determined using data from 3 independent experiments (**, p < .01; ****, p < .0001). (TIF 55 kb) [file 13100_2019_159_MOESM5_ESM.tif]

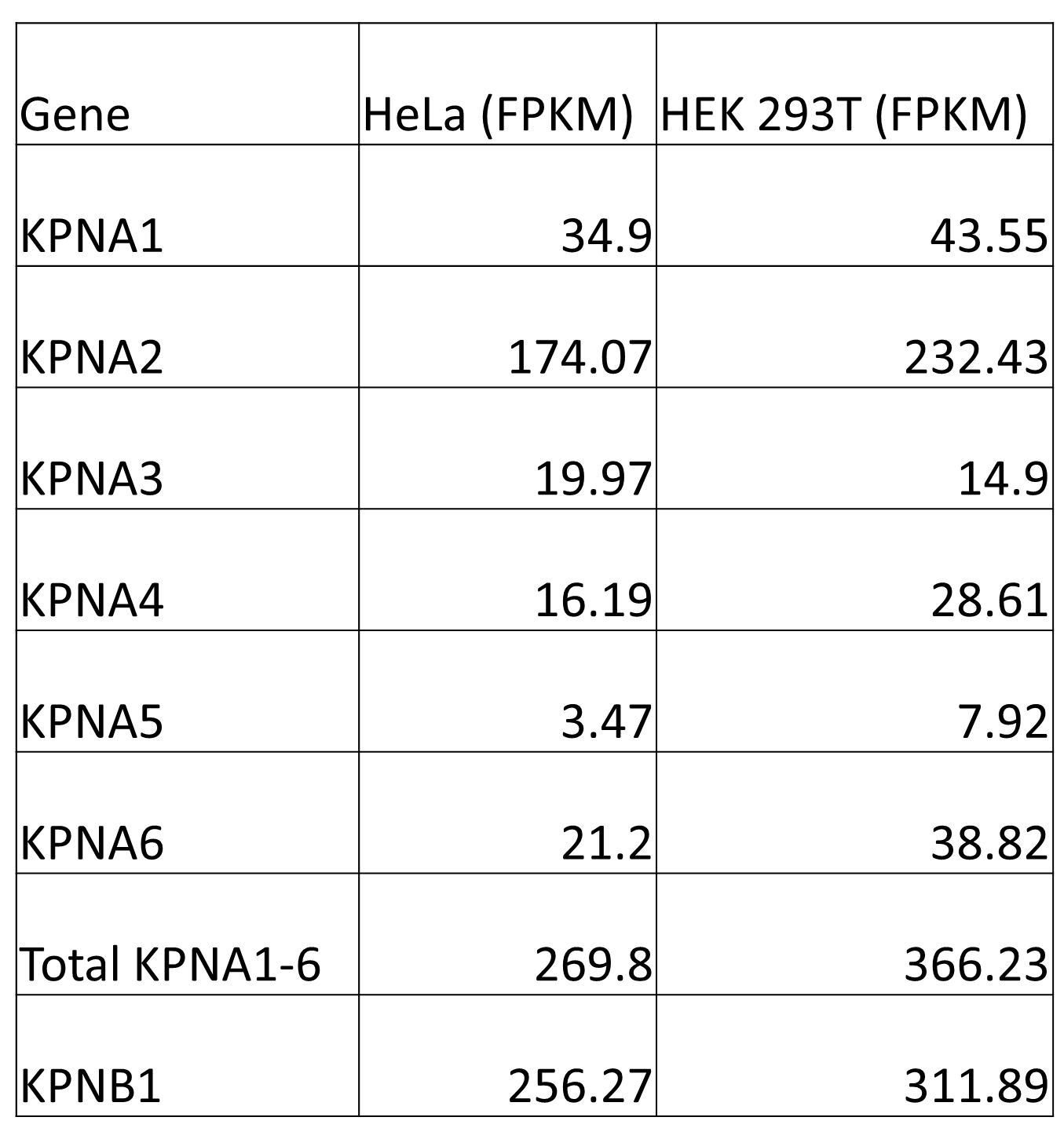

Supplement: Supplementary file 6 — Table S1. Expression profile of import genes in HEK293T cells and HeLa cells. Expression profiles for HeLa cells were determined using RNA-seq dataset79. Expression profiles for HEK293T cells were determined using RNA-seq data publically available through NCBI SRA (SRR1182596). Data calculated as fragments per kilobase of transcript per million mapped reads (FPKM). (TIF 140 kb) [file 13100_2019_159_MOESM6_ESM.tif]
