# Supplementary material for: Identification of charged amino acids required for nuclear localization of human L1 ORF1 protein
Source: Mob DNA. 2019 May 6;10:20. doi: 10.1186/s13100-019-0159-2 (PMC6501352; doi:10.1186/s13100-019-0159-2)
Supplement: Supplementary file 7 — Alignment of ORF1 sequences from genomic L1s. Alignment performed using clustal W method relative to the human ORF1p L1PA1 sequence. (PDF 75 kb) [file 13100_2019_159_MOESM7_ESM.pdf]

- Majority

```
Majority      MGKKQNRKTGNSKTQSASPPPKERSSSPATEQSWMENDFDELREEGFRRSNYSELREDIQTKGKEVENFE
                       10        20        30        40        50        60        70
ORF1 L1.3     MGKKQNRKTGNSKTQSASPPPKERSSSPATEQSWMENDFDELREEGFRRSNYSELREDIQTKGKEVENFE  70
Chr1.seq97    ......................................................................  70
Chr1.seq98    ......................................................................  70
Chr1.seq99    ......................................................................  70
Chr1.seq100   ..........................................S...........................  70
Chr1.seq101   ................T......S..............................................  70
Chr1.seq102   .............M.............L..........................................  70
Chr1.seq103   ......................................................................  70
Chr1.seq104   ......................................................................  70
Chr1.seq105   ......................................................................  70
Chr1.seq106   ......................................................................  70
Chr1.seq107   ......................................................................  70
Chr1.seq108   ................V.......................K.............................  70
Chr1.seq109   ......................................................................  70
Chr1.seq110   ......................................................................  70
Chr1.seq111   ................T.....................................................  70
Chr2.seq40    .............K........................................................  70
Chr2.seq41    ..................................................D...................  70
Chr2.seq42    .......................................................Q..............  70
Chr2.seq43    ........................................K.............................  70
Chr2.seq44    .............M........................................................  70
Chr2.seq45    ................T.............D.......................................  70
Chr2.seq46    ......................................................................  70
Chr2.seq47    ........................R.............................................  70
Chr2.seq48    ......................................................................  70
Chr2.seq49    ................T............................V..Q.....................  70
Chr3.seq75    ...........................T...........................W..............  70
Chr3.seq76    ...........C............................................G.............  70
Chr3.seq77    .......................................................Q..............  70
Chr3.seq78    ......................................................................  70
Chr3.seq79    ......................................................................  70
Chr3.seq82    .............M...................C....................................  70
Chr3.seq83    ......................................................................  70
Chr3.seq84    .......................C..............................................  70
Chr4.seq23    ......................................................................  70
Chr4.seq24    .......................................................Q..............  70
Chr4.seq25    ......................................................................  70
Chr4.seq26    ......................................................................  70
Chr4.seq27    .............M..................................Q.....................  70
Chr4.seq28    ......................................................................  70
Chr4.seq29    ......................................................................  70
Chr4.seq30    .V...............................................................A....  70
Chr4.seq31    .........................................................E............  70
Chr4.seq32    ...........................L..........................................  70
Chr5.seq55    ................................................Q.....................  70
Chr5.seq56    .....K.....................L..........................................  70
```

Chr5.seq57	...........P..	70
Chr5.seq58	....	70
Chr5.seq59	....	70
Chr5.seq60	....D....W..............	70
Chr5.seq61	....	70
Chr5.seq63	....	70
Chr5.seq64	....	70
Chr5.seq65	..........S..N.........C...............................W..............	70
Chr5.seq66	.............M..	70
Chr6.seq112	....	70
Chr6.seq113	....	70
Chr6.seq114	....K.............................	70
Chr6.seq115	....	70
Chr6.seq116	....	70
Chr6.seq117	.........................F..............V.............................	70
Chr6.seq118	....E........	70
Chr6.seq119	....	70
Chr6.seq121	....	70
Chr7.seq67	....	70
Chr7.seq68	....	70
Chr7.seq69	....	70
Chr7.seq70	....	70
Chr7.seq71	....R...K...................	70
Chr7.seq72	....	70
Chr7.seq74	....	70
Chr8.seq1	....	70
Chr8.seq2	..........S.EN.......R............V...................................	70
Chr8.seq3	....	70
Chr8.seq4	....Q..............	70
Chr8.seq5	....	70
Chr8.seq6	....	70
Chr9.seq122	....	70
Chr9.seq123	.............M..	70
Chr9.seq125	.............K..	70
Chr10.seq34	....	70
Chr10.seq35	................V...	70
Chr10.seq36	.................Y.....H..	70
Chr10.seq37	....	70
Chr10.seq38	....	70
Chr10.seq39	....	70
Chr11.seq8	....	70
Chr11.seq9	.......................H..K.....	70
Chr11.seq10	....	70
Chr11.seq11	....N.........	70
Chr11.seq12	....	70
Chr11.seq13	....	70
Chr11.seq14	....	70
Chr11.seq15	....	70
Chr12.seq50	....	70
Chr12.seq51	..........S..N...............M..T.....................................	70
Chr12.seq52	................V......H.....M..	70
Chr12.seq53	........................C...	70

```
Chr12.seq54    ......................................................................  70
Chr13.seq126   .............K....................T...................................  70
Chr13.seq127   ..........S..N.........................................Q..V...........  70
Chr13.seq128   ......................................................................  70
Chr14.seq138   .............................M........................................  70
Chr14.seq139   ......................................................................  70
Chr14.seq140   .................................................................L....  70
Chr15.seq133   ......................................................................  70
Chr15.seq134   ................................................Q.....................  70
Chr15.seq135   ......................................................................  70
Chr15.seq136   .............I..V.....................................................  70
Chr15.seq137   ................T.....................................................  70
Chr16.seq16    .............K..T...............N..........................E..........  70
Chr16.seq17    ..................................L...................................  70
Chr16.seq18    ......................................................................  70
Chr16.seq19    .............K..T....................G.................W..............  70
Chr16.seq20    ...........................L..........................................  70
Chr16.seq21    ......................................................................  70
Chr16.seq22    ......................................................................  70
Chr17.seq33    ..................................................S...................  70
Chr18.seq141   ......................................................................  70
Chr18.seq142   ......................................................................  70
Chr18.seq143   ......................................................................  70
Chr18.seq144   .................................C....................................  70
Chr18.seq145   .............K.........H................K.............................  70
Chr20.seq131   ......................................................................  70
Chr20.seq132   ......................................................................  70
Chr22.seq129   .................................................................I....  70
Chr22.seq130   ......................................................................  70
ChrX.seq85     ......................................................................  70
ChrX.seq86     ......................................................................  70
ChrX.seq88     ......................................................................  70
ChrX.seq89     .............M.............L..........................................  70
ChrX.seq90     ......................................................................  70
ChrX.seq91     ......................................................................  70
ChrX.seq92     .............................M........................................  70
ChrX.seq96     ......................................................................  70
```

- Majority

```
Majority       KNLEECITRITNTEKCLKELMELKTKARELREECRSLRSRCDQLEERVSAMEDEMNEMKREGKFREKRIK
                        80        90        100       110       120       130       140
ORF1 L1.3      KNLEECITRITNTEKCLKELMELKTKARELREECRSLRSRCDQLEERVSAMEDEMNEMKREGKFREKRIK  140
Chr1.seq97     ..............................................S.......................  140
Chr1.seq98     ......................................................................  140
Chr1.seq99     ......................................................................  140
Chr1.seq100    ......................................................................  140
Chr1.seq101    ......................................................................  140
Chr1.seq102    ...............................................................V......  140
Chr1.seq103    ......................................................................  140
```

```
Chr1.seq104  ......................................................................  140
Chr1.seq105  ..............................H........Q..........................K....  140
Chr1.seq106  ......................................................................  140
Chr1.seq107  ......................................................................  140
Chr1.seq108  ......................................................................  140
Chr1.seq109  ......................................................................  140
Chr1.seq110  ......................................................................  140
Chr1.seq111  ......................................................................  140
Chr2.seq40   ..............N....V...................Q..........T...................  140
Chr2.seq41   ...........................................................Q..........  140
Chr2.seq42   T.....................................................................  140
Chr2.seq43   ......................................................................  140
Chr2.seq44   ......................P...............................................  140
Chr2.seq45   .......................................Q...................Q..........  140
Chr2.seq46   ...........................Q..............................N....V......  140
Chr2.seq47   ......................................................................  140
Chr2.seq48   ..............................C............................Q..........  140
Chr2.seq49   ......................................................................  140
Chr3.seq75   .........................................H............................  140
Chr3.seq76   ....................................P.................................  140
Chr3.seq77   ......................................................................  140
Chr3.seq78   ......................................................................  140
Chr3.seq79   .........................................N....K.......................  140
Chr3.seq82   ......................................................................  140
Chr3.seq83   ......................................................................  140
Chr3.seq84   .......................................Q..............................  140
Chr4.seq23   ......................................................................  140
Chr4.seq24   ......................................................................  140
Chr4.seq25   ......................................................................  140
Chr4.seq26   ......................................................................  140
Chr4.seq27   ...........................................................L..........  140
Chr4.seq28   ......................................................................  140
Chr4.seq29   ......................................................................  140
Chr4.seq30   .............................................G........................  140
Chr4.seq31   ......................................................................  140
Chr4.seq32   ...............................................................V......  140
Chr5.seq55   ......................................................................  140
Chr5.seq56   .................................................V....................  140
Chr5.seq57   ......................................................................  140
Chr5.seq58   ......................................................................  140
Chr5.seq59   ......................................................................  140
Chr5.seq60   ......................................................................  140
Chr5.seq61   .........................................N............................  140
Chr5.seq63   ......................................................................  140
Chr5.seq64   ......................................................................  140
Chr5.seq65   .K...............................................V....................  140
Chr5.seq66   ......................................................................  140
Chr6.seq112  ......................................................................  140
Chr6.seq113  .............................V..K..........................Q..........  140
Chr6.seq114  .......................................Q.......................V......  140
Chr6.seq115  ...........................Q..H.......................................  140
Chr6.seq116  ......................................................................  140
```

Chr6.seq117	. . . . V	140
Chr6.seq118	. . . .	140
Chr6.seq119	. . . .	140
Chr6.seq121	. . . . N	140
Chr7.seq67	. . . .	140
Chr7.seq68	. . . .	140
Chr7.seq69	. . . . E	140
Chr7.seq70	. . . .	140
Chr7.seq71	. . . .	140
Chr7.seq72	. . . . R	140
Chr7.seq74	. . . .	140
Chr8.seq1	. . . .	140
Chr8.seq2	. . . . K . . . . G	140
Chr8.seq3	. . . .	140
Chr8.seq4	. . . .	140
Chr8.seq5	. . . .	140
Chr8.seq6	. . . .	140
Chr9.seq122	. . . .	140
Chr9.seq123	. . . . G	140
Chr9.seq125	. . . . H	140
Chr10.seq34	. . . .	140
Chr10.seq35	. . . . N	140
Chr10.seq36	. . . . Q . . P . . . . V	140
Chr10.seq37	. . . .	140
Chr10.seq38	. . . .	140
Chr10.seq39	. . . .	140
Chr11.seq8	. . . .	140
Chr11.seq9	. . . . Q . . . . N	140
Chr11.seq10	. . . .	140
Chr11.seq11	. . . .	140
Chr11.seq12	. . . .	140
Chr11.seq13	. . . . N	140
Chr11.seq14	. . . .	140
Chr11.seq15	. . . .	140
Chr12.seq50	. . . .	140
Chr12.seq51	. . . . N	140
Chr12.seq52	. . . . N	140
Chr12.seq53	. . . . Q	140
Chr12.seq54	. . . .	140
Chr13.seq126	. . . . T	140
Chr13.seq127	. . . . H . . . . W	140
Chr13.seq128	. . . .	140
Chr14.seq138	. . . .	140
Chr14.seq139	. . . .	140
Chr14.seq140	. . . . H . . . . Q	140
Chr15.seq133	. . . .	140
Chr15.seq134	. . . .	140
Chr15.seq135	. . . .	140
Chr15.seq136	. . . . C . . . . Q	140
Chr15.seq137	. . . .	140
Chr16.seq16	. . . . T . . . . V	140
Chr16.seq17	. . . .	140

```
Chr16.seq18    ..........S...........................................................  140
Chr16.seq19    ...............................................................S......  140
Chr16.seq20    .........................................N............................  140
Chr16.seq21    ......................................................................  140
Chr16.seq22    ......................................................................  140
Chr17.seq33    ...............Y......................................................  140
Chr18.seq141   .........................................N............................  140
Chr18.seq142   ...................................T..................................  140
Chr18.seq143   ......................................................................  140
Chr18.seq144   ......................................................................  140
Chr18.seq145   ......................................................................  140
Chr20.seq131   ......................................................................  140
Chr20.seq132   ......................................................................  140
Chr22.seq129   ......................................................................  140
Chr22.seq130   ..........................T...........................................  140
ChrX.seq85     ......................................................................  140
ChrX.seq86     ........................................W.............................  140
ChrX.seq88     ......................................................................  140
ChrX.seq89     ......................................................................  140
ChrX.seq90     .................................................................D....  140
ChrX.seq91     ......................................................................  140
ChrX.seq92     ...........................................V...............Q..........  140
ChrX.seq96     ......................................................................  140
```

- Majority

```
Majority       RNEQSLQEIWDYVKRPNLRLIGVPESDVENGTKLENTLQDIIQENFPNLARQANVQIQEIQRTPQRYSSR
                         150       160       170       180       190       200       210
ORF1 L1.3      RNEQSLQEIWDYVKRPNLRLIGVPESDVENGTKLENTLQDIIQENFPNLARQANVQIQEIQRTPQRYSSR  210
Chr1.seq97     .......K..........C.T.................................................  210
Chr1.seq98     ..................................................................F...  210
Chr1.seq99     ......................................................................  210
Chr1.seq100    ......................................................................  210
Chr1.seq101    .........................................T............................  210
Chr1.seq102    ......................................................................  210
Chr1.seq103    ......................................................................  210
Chr1.seq104    ...........................G..........................................  210
Chr1.seq105    ......................................................................  210
Chr1.seq106    ......................................................................  210
Chr1.seq107    ........................Q.............................................  210
Chr1.seq108    ...........................GD.........................................  210
Chr1.seq109    ......................................................................  210
Chr1.seq110    ......................................................................  210
Chr1.seq111    ......................................................................  210
Chr2.seq40     ...........................GK.......S.................................  210
Chr2.seq41     .K.........................G..........................................  210
Chr2.seq42     ......................................................................  210
Chr2.seq43     ....N.....V..............R............................................  210
Chr2.seq44     ......................................................................  210
Chr2.seq45     ...........................A................T.........................  210
```

Chr2.seq46	. . . . A . . . . I . . . . . H	210
Chr2.seq47	. . . . T	210
Chr2.seq48	. . . . M	210
Chr2.seq49	. . . . M	210
Chr3.seq75	. . . .	210
Chr3.seq76	. . . . G . . . . I	210
Chr3.seq77	. . . .	210
Chr3.seq78	. . . .	210
Chr3.seq79	. . . .	210
Chr3.seq82	. . . . I . . . . M	210
Chr3.seq83	. . . .	210
Chr3.seq84	. . . .	210
Chr4.seq23	. . . .	210
Chr4.seq24	. . . .	210
Chr4.seq25	. . . . T	210
Chr4.seq26	. . . .	210
Chr4.seq27	. . . .	210
Chr4.seq28	. . . . K	210
Chr4.seq29	. . . .	210
Chr4.seq30	. . . . H	210
Chr4.seq31	. . . .	210
Chr4.seq32	. . . .	210
Chr5.seq55	. . . . F . . .	210
Chr5.seq56	. . . .	210
Chr5.seq57	. . . .	210
Chr5.seq58	. . . .	210
Chr5.seq59	. . . .	210
Chr5.seq60	. . . . R	210
Chr5.seq61	. . . .	210
Chr5.seq63	. . . .	210
Chr5.seq64	. . . .	210
Chr5.seq65	. . . . G . . . . I . . . . C . . .	210
Chr5.seq66	. . . . I	210
Chr6.seq112	. . . .	210
Chr6.seq113	. . . . A . . . . S . . . . L .	210
Chr6.seq114	. H . . . . A . . . . S	210
Chr6.seq115	. . . . S	210
Chr6.seq116	. . . . E	210
Chr6.seq117	. . . . A	210
Chr6.seq118	. . . .	210
Chr6.seq119	. . . . I	210
Chr6.seq121	. . . . G	210
Chr7.seq67	. . . .	210
Chr7.seq68	. . . .	210
Chr7.seq69	. . . . N	210
Chr7.seq70	. . . .	210
Chr7.seq71	. . . .	210
Chr7.seq72	. . . . M	210
Chr7.seq74	. . . .	210
Chr8.seq1	. . . .	210
Chr8.seq2	. . . . H . . . L . . . . G . . . . L	210
Chr8.seq3	. . . .	210

```
Chr8.seq4       ......................................................................  210
Chr8.seq5       ......................................................................  210
Chr8.seq6       ......................................................................  210
Chr9.seq122     ......................................................................  210
Chr9.seq123     ......................................................................  210
Chr9.seq125     ...........................G..................................M.......  210
Chr10.seq34     ..............................................................M.......  210
Chr10.seq35     ......................................................................  210
Chr10.seq36     ..................H........A..........................................  210
Chr10.seq37     ......................................................................  210
Chr10.seq38     ......................................................................  210
Chr10.seq39     ......................................................................  210
Chr11.seq8      ....N.................................................................  210
Chr11.seq9      ........................K..G..........................................  210
Chr11.seq10     ....................T.................................................  210
Chr11.seq11     ....................T.................................................  210
Chr11.seq12     ......................................................................  210
Chr11.seq13     ...........................G..........................................  210
Chr11.seq14     ......................................................I...............  210
Chr11.seq15     ......................................................................  210
Chr12.seq50     ..A...................................................................  210
Chr12.seq51     ...E..................................................................  210
Chr12.seq52     ..Q........................G..............................K...........  210
Chr12.seq53     ......................................................................  210
Chr12.seq54     ......................................................................  210
Chr13.seq126    ...........................A.......D.......................T..........  210
Chr13.seq127    ...........................G..................................M.......  210
Chr13.seq128    ......................................................................  210
Chr14.seq138    .................P.........G........................................L.  210
Chr14.seq139    ......................................................................  210
Chr14.seq140    ............L..............G........................................L.  210
Chr15.seq133    ......................................................................  210
Chr15.seq134    ......................................................................  210
Chr15.seq135    ......................................................................  210
Chr15.seq136    ..................P........G............T.....................M.......  210
Chr15.seq137    ......................................................................  210
Chr16.seq16     ...........................G.................V......................L.  210
Chr16.seq17     ......................................................................  210
Chr16.seq18     ......................................................................  210
Chr16.seq19     ...........................G..................................M.......  210
Chr16.seq20     ......................................................................  210
Chr16.seq21     .....................................................DI...............  210
Chr16.seq22     ......................................................................  210
Chr17.seq33     ....................................................................L.  210
Chr18.seq141    ......................................................................  210
Chr18.seq142    ......................................................................  210
Chr18.seq143    ...........................G..........................................  210
Chr18.seq144    ...........................G...................................S......  210
Chr18.seq145    ...........................G..........................................  210
Chr20.seq131    ......................................................................  210
Chr20.seq132    ......................................................................  210
Chr22.seq129    ...........................................................V..........  210
```

```
Chr22.seq130  ......................................................................  210
ChrX.seq85    ......................................................................  210
ChrX.seq86    ....................T...........T.....................................  210
ChrX.seq88    ......................................................................  210
ChrX.seq89    ......................................................................  210
ChrX.seq90    ......................................................................  210
ChrX.seq91    ...........................E..........................................  210
ChrX.seq92    ......................................................................  210
ChrX.seq96    ......................................................................  210
```

- Majority

```
Majority      RATPRHIIVRFTKVEMKEKMLRAAREKGRVTLKGKPIRLTADLSAETLQARREWGPIFNILKEKNFQPRI
                       220       230       240       250       260       270       280

ORF1 L1.3     RATPRHIIVRFTKVEMKEKMLRAAREKGRVTLKGKPIRLTVDLSAETLQARREWGPIFNILKEKNFQPRI  280
Chr1.seq97    ........................................A.............................  280
Chr1.seq98    ...................I....................A.............................  280
Chr1.seq99    ........................................A.............................  280
Chr1.seq100   ........................................A.............................  280
Chr1.seq101   ........................................A.....................K.....I.  280
Chr1.seq102   ...A....................................A.............................  280
Chr1.seq103   ........................................A.............................  280
Chr1.seq104   ..............................P.........A.............................  280
Chr1.seq105   ........................................A.............R...............  280
Chr1.seq106   ........................................A.............................  280
Chr1.seq107   ........................................A.............................  280
Chr1.seq108   ........................................A.............................  280
Chr1.seq109   ........................................A.............................  280
Chr1.seq110   ........................................A.............................  280
Chr1.seq111   ........................................A.............................  280
Chr2.seq40    .......................................AA..L..........................  280
Chr2.seq41    ........................................A.............................  280
Chr2.seq42    ........................................A.............................  280
Chr2.seq43    ........................................A.............................  280
Chr2.seq44    ........................................A.............................  280
Chr2.seq45    ........................................A................L............  280
Chr2.seq46    ........................................A.I....V......................  280
Chr2.seq47    ........................................A.............................  280
Chr2.seq48    ............................Q...........A.............................  280
Chr2.seq49    ........................................A.............................  280
Chr3.seq75    ....................F...................A.............................  280
Chr3.seq76    ..............Q.........................A..L..........................  280
Chr3.seq77    G.......................................A.............................  280
Chr3.seq78    ........................................A.............................  280
Chr3.seq79    ........................................A.............................  280
Chr3.seq82    ........................................A.............................  280
Chr3.seq83    ........................................A.............................  280
Chr3.seq84    ........................................A.............................  280
Chr4.seq23    ........................................A.............................  280
Chr4.seq24    ........................................A.............................  280
```

Chr4.seq25	....A.....................G.......	280
Chr4.seq26	....A..L.............Y............	280
Chr4.seq27	....A.............................	280
Chr4.seq28	....A.............................	280
Chr4.seq29	....A.............................	280
Chr4.seq30	....A.............................	280
Chr4.seq31	....A.............................	280
Chr4.seq32	....A.............................	280
Chr5.seq55	....A.............................	280
Chr5.seq56	....A.............................	280
Chr5.seq57	....A.............................	280
Chr5.seq58	....A.............................	280
Chr5.seq59	....A.............................	280
Chr5.seq60	...R....................................A.........G...................	280
Chr5.seq61	...................T....................A.............................	280
Chr5.seq63	.......................T................A.............................	280
Chr5.seq64	....A.............................	280
Chr5.seq65	....A.............................	280
Chr5.seq66	....A.............................	280
Chr6.seq112	....A.............................	280
Chr6.seq113	......................T.................A.............................	280
Chr6.seq114	....A.............................	280
Chr6.seq115	..I.....................................A.............................	280
Chr6.seq116	....A.............................	280
Chr6.seq117	......................E.................A........T....................	280
Chr6.seq118	....	280
Chr6.seq119	.......................T................A.............................	280
Chr6.seq121	....A.............................	280
Chr7.seq67	...........................V............A.............................	280
Chr7.seq68	......................................I.A.............................	280
Chr7.seq69	.............L..........................A.............................	280
Chr7.seq70	....A.............................	280
Chr7.seq71	....A.............................	280
Chr7.seq72	....A.............................	280
Chr7.seq74	....A.............................	280
Chr8.seq1	....A.............................	280
Chr8.seq2	....A.............................	280
Chr8.seq3	....A.............................	280
Chr8.seq4	....A.............................	280
Chr8.seq5	....A.............................	280
Chr8.seq6	....	280
Chr9.seq122	.......M................................A.............................	280
Chr9.seq123	....A.............................	280
Chr9.seq125	............................Q...........A.....................D.......	280
Chr10.seq34	....A.............................	280
Chr10.seq35	....A.............................	280
Chr10.seq36	.......................V................A.............................	280
Chr10.seq37	....A.............................	280
Chr10.seq38	.....................K..................A.............................	280
Chr10.seq39	........I...............................A.............................	280
Chr11.seq8	....A...................V.........	280
Chr11.seq9	....	280

```
Chr11.seq10    ......................................................................  280
Chr11.seq11    ........................................A.............................  280
Chr11.seq12    ........................................A.............................  280
Chr11.seq13    ........................................A....K........................  280
Chr11.seq14    ........................................A.................I...........  280
Chr11.seq15    ........................................A.............................  280
Chr12.seq50    ........................................A..........................S..  280
Chr12.seq51    ............................Q...........A..L..........................  280
Chr12.seq52    ........................................A...S.........................  280
Chr12.seq53    ........................................A........T.....T..............  280
Chr12.seq54    ........................................A.............................  280
Chr13.seq126   ...................................................................R..  280
Chr13.seq127   ........................................A.............................  280
Chr13.seq128   ........................................A.............................  280
Chr14.seq138   ....................................M...A.............................  280
Chr14.seq139   ........................................A.............................  280
Chr14.seq140   ........................................A.............................  280
Chr15.seq133   ........................................A.............................  280
Chr15.seq134   ......................................................................  280
Chr15.seq135   ............................Q...........A.............................  280
Chr15.seq136   ........................................A.............................  280
Chr15.seq137   ........................................A.............................  280
Chr16.seq16    ..........................E................L..........................  280
Chr16.seq17    ........................................A.............................  280
Chr16.seq18    ........................................A.............................  280
Chr16.seq19    ...................................RV.V.A.............................  280
Chr16.seq20    ........................................A.............................  280
Chr16.seq21    ........................................A.............................  280
Chr16.seq22    ........................................A.....................D.......  280
Chr17.seq33    ........................................A.............................  280
Chr18.seq141   ........................................A.............................  280
Chr18.seq142   ......................E.................A.............................  280
Chr18.seq143   ........................................A.............................  280
Chr18.seq144   ........................................A.............................  280
Chr18.seq145   ...............................P........A.............................  280
Chr20.seq131   ........................................A.............................  280
Chr20.seq132   ........................................A.............................  280
Chr22.seq129   ........................................A.............................  280
Chr22.seq130   ........................................A.............................  280
ChrX.seq85     ........................................A.............................  280
ChrX.seq86     .........................D..............A.............................  280
ChrX.seq88     ........................................A.............................  280
ChrX.seq89     ........................................A.............................  280
ChrX.seq90     ........................................A.............................  280
ChrX.seq91     ........................................A.............................  280
ChrX.seq92     ........................................A.............................  280
ChrX.seq96     ........................................A.............................  280
```

- Majority

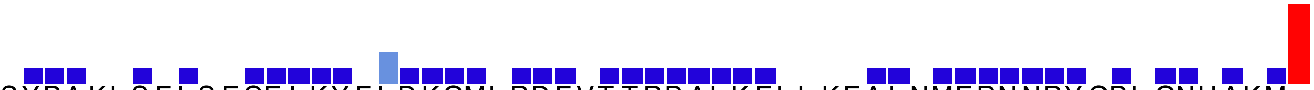

```
Majority       SYPAKLSFISEGEIKYFIDKQMLRDFVTTRPALKELLKEALNMERNNRYQPLQNHAKM-
```

```
                      290       300       310       320       330
                        |         |         |         |         |
ORF1 L1.3      SYPAKLSFISEGEIKYFIDKQMLRDFVTTRPALKELLKEALNMERNNRYQPLQNHAKM.  339
Chr1.seq97     ..........................................................   338
Chr1.seq98     .......................................V..................   338
Chr1.seq99     .F...........R...T.............T..........................   338
Chr1.seq100    ..........................................................   338
Chr1.seq101    ...........................N...................W..........   338
Chr1.seq102    .......................................S..................   338
Chr1.seq103    ..........................................................   338
Chr1.seq104    ...............................................W..........   338
Chr1.seq105    ..........................................................   338
Chr1.seq106    ..........................................................   338
Chr1.seq107    ..........................................................   338
Chr1.seq108    .................T.....T..................................   338
Chr1.seq109    .F...........R...T.............T..........................   338
Chr1.seq110    ..........................................................   338
Chr1.seq111    ..................E...............................L.......   338
Chr2.seq40     .................T...........T............T.K.............   338
Chr2.seq41     .................T.....................V..................   338
Chr2.seq42     ..........................................................   338
Chr2.seq43     ..........................................................   338
Chr2.seq44     ...D......................................................   338
Chr2.seq45     .................T.....T....S.............................   338
Chr2.seq46     .................T.............................W..........   338
Chr2.seq47     ..........................................................   338
Chr2.seq48     ..........................................................   338
Chr2.seq49     ..........................................................   338
Chr3.seq75     ..............................H...........................   338
Chr3.seq76     .................T................................L.......   338
Chr3.seq77     ..........................................................   338
Chr3.seq78     .............................T............................   338
Chr3.seq79     ..........................................................   338
Chr3.seq82     ..........................................................   338
Chr3.seq83     ..........................................................   338
Chr3.seq84     ..........................................L..............I   338
Chr4.seq23     ..........................................................   338
Chr4.seq24     ..........................................................   338
Chr4.seq25     .....................................................S....   338
Chr4.seq26     ..........................................................   338
Chr4.seq27     ..........................................................   338
Chr4.seq28     ..T.......................................................   338
Chr4.seq29     ..........................................................   338
Chr4.seq30     .......................................V..................   338
Chr4.seq31     ..........................................................   338
Chr4.seq32     .......................................S..................   338
Chr5.seq55     .......................................V..................   338
Chr5.seq56     ..........................................................   338
Chr5.seq57     ...............................................W..........   338
Chr5.seq58     ..........................................................   338
Chr5.seq59     ..........................................................   338
Chr5.seq60     ..........................................................   338
```

Chr5.seq61	. . . .	338
Chr5.seq63	. . . .	338
Chr5.seq64	. F . . . . A . . . . . T . . . . T	338
Chr5.seq65	. . . . T . . . . P . . . . Q	338
Chr5.seq66	. . . . I	338
Chr6.seq112	. . . . . . N . . . . V . . . . D . .	338
Chr6.seq113	. . . . T . . . . Q H	338
Chr6.seq114	. . . . T . . K	338
Chr6.seq115	. . . .	338
Chr6.seq116	. . . .	338
Chr6.seq117	. . . . T . . . . V . . . . . K . W	338
Chr6.seq118	. . . .	338
Chr6.seq119	. . . .	338
Chr6.seq121	. . . .	338
Chr7.seq67	. . . . V	338
Chr7.seq68	. . . . . . T	338
Chr7.seq69	. . . .	338
Chr7.seq70	. . . .	338
Chr7.seq71	. . . . T	338
Chr7.seq72	. . . . K	338
Chr7.seq74	. . . .	338
Chr8.seq1	. . . . Q	338
Chr8.seq2	. . . . L . . . . R . . . T . . . . M . . . . I	338
Chr8.seq3	. H	338
Chr8.seq4	. . . .	338
Chr8.seq5	. . . . W	338
Chr8.seq6	. . . .	338
Chr9.seq122	. . . .	338
Chr9.seq123	. . . . Q	338
Chr9.seq125	. . . G	338
Chr10.seq34	. . . .	338
Chr10.seq35	. . . . V	338
Chr10.seq36	. . . . T . . . . W	338
Chr10.seq37	. . . .	338
Chr10.seq38	. . . . T	338
Chr10.seq39	. . . .	338
Chr11.seq8	. . . .	338
Chr11.seq9	. . . .	338
Chr11.seq10	. . . .	338
Chr11.seq11	. . . .	338
Chr11.seq12	. . . .	338
Chr11.seq13	. . . .	338
Chr11.seq14	. . . .	338
Chr11.seq15	. . . . V	338
Chr12.seq50	. . . . Q	338
Chr12.seq51	. . . . T . . . . Q	338
Chr12.seq52	. . . . T . . . . Q	338
Chr12.seq53	. . . .	338
Chr12.seq54	. . . .	338
Chr13.seq126	. . A . . . . T . . . . Q	338
Chr13.seq127	. . . . T . . . . Q . . L	338
Chr13.seq128	. . . .	338

Chr14.seq138	. . . .	338
Chr14.seq139	. . . .	338
Chr14.seq140	. . . . T . . . V	338
Chr15.seq133	. . T	338
Chr15.seq134	. . . . S . T	338
Chr15.seq135	. . . . L . . . . D	338
Chr15.seq136	. . . . T . . . . . T . . . . . K	338
Chr15.seq137	. . . . R . . . T . . . . T	338
Chr16.seq16	. . . . T . . . . T . . . . Q . . . . L	338
Chr16.seq17	. . . .	338
Chr16.seq18	. . . .	338
Chr16.seq19	. . . . T . . . . . . N . . . . Q . . . . R	338
Chr16.seq20	. . . . M	338
Chr16.seq21	. . . . R . . . T . . . . T	338
Chr16.seq22	. . . . V . . . . T	338
Chr17.seq33	. . . . T . . . . L	338
Chr18.seq141	. . . .	338
Chr18.seq142	. . . . L	338
Chr18.seq143	. . . .	338
Chr18.seq144	. . . . H	338
Chr18.seq145	. . . . . . N . . . . T	338
Chr20.seq131	. . . . T	338
Chr20.seq132	. . . . S . T . R	338
Chr22.seq129	. . . .	338
Chr22.seq130	. . . .	338
ChrX.seq85	. . . . Q	338
ChrX.seq86	. . . . N	338
ChrX.seq88	. . . . V	338
ChrX.seq89	. . . . K . . . . S	338
ChrX.seq90	. . . .	338
ChrX.seq91	. . . . N . . . . I . . . . L	338
ChrX.seq92	. . . . D	338
ChrX.seq96	. . . .	338

Decoration 'Decoration #1': Hide (as '.') residues that match ORF1 L1.3 exactly.
